# A neural substrate for sensory over-responsivity defined by exogenous and endogenous brain systems

**DOI:** 10.1186/s11689-025-09656-y

**Published:** 2025-11-21

**Authors:** Hannah L. Choi, Maia C. Lazerwitz, Rachel Powers, Mikaela Rowe, Jamie Wren-Jarvis, Amir Sadikov, Lanya T. Cai, Robyn Chu, LaShelle Rullan, Kaitlyn J. Trimarchi, Rafael D. Garcia, Elysa J. Marco, Pratik Mukherjee

**Affiliations:** 1 Department of Radiology & Biomedical Imaging, University of California, UCSF, San Francisco, 94143 USA; 2 Department of Neurological Surgery, University of California, UCSF, San Francisco, 94143 USA; 3 Cortica Healthcare, San Rafael, 94903 USA; 4 Vilcek Institute of Graduate Biomedical Sciences, New York University, New York, 10016 USA; 5 Joint Graduate Program in Bioengineering, University of California, UC Berkeley and UCSF, Berkeley, 94720 USA; 6 Growing Healthy Seasons Therapy Services, Rescue, 95672 USA; 7 Lifetime Neurodevelopmental Care Center, Encino, 91316 USA

**Keywords:** Sensory over-responsivity, Sensory processing disorder, Neurodevelopment, Functional MRI connectivity, Multimodal neuroimaging, Emotional regulation, Resilience

## Abstract

**Background:**

Exogenous (outward-directed) and endogenous (inward-directed) neural systems are essential for cognition and behavior. However, how they are altered in neurodiverse (ND) children remains unanswered in part due to heterogeneity. Sensory over-responsivity (SOR), the most prevalent form of sensory processing disorder (SPD), serves as a quintessential paradigm for investigating the interaction between exogenous and endogenous brain networks given that both basic and higher-order sensory processing are substantially implicated in this condition.

**Methods:**

Neurodiverse children ages 8–12 years old (*n* = 83; 30 females and 53 males) were directly assessed for SOR using a structured clinical evaluation, the Sensory Processing 3 Dimensions Assessment (SP3D:A), and underwent 3 Tesla MRI. 39 ND children presented with SOR (ND/SOR) and 44 ND children presented without SOR (ND/NO-SOR). Exogenous and endogenous functional connectivity networks (FCNs) were generated through independent component analysis and investigated with two local functional connectivity (FC) measures, fractional amplitude of low-frequency fluctuations (fALFF) and regional homogeneity (ReHo), as well as a long-range FC measure, dual regression (DR). Moreover, we examined FC in the context of behavioral regulation as assessed through the Behavioral Assessment System for Children, 3rd edition (BASC-3), categorizing children as “resilient” or “dysregulated” through latent profile analysis.

**Results:**

In general, ND/SOR children exhibit reduced long-range exogenous FC. However, in terms of local FC, we find that ND/SOR children have reduced exogenous and elevated endogenous FC which is diametrically opposed to ND/NO-SOR children. Furthermore, this double dissociation is specific to ND children who are behaviorally resilient, while emotionally dysregulated ND children possess a distinct pattern.

**Conclusion:**

Achieving optimal brain system connectivity—a balanced contrast—is influenced by sensory over-responsivity and essential for resilience.

**Supplementary Information:**

The online version contains supplementary material available at 10.1186/s11689-025-09656-y.

## Background

Human survival depends on the proper cohesion of brain systems responsible for predicting and processing incoming sensory information, filtering and integrating information in the context of the environment and previous experience, and planning and executing an appropriate, well-modulated response. Recent work describes a sensorimotor–association (SA) axis for the human cerebral cortex with reciprocal connections between the unimodal sensory/motor regions that are largely posteriorly located in frontal, parietal, temporal, and occipital lobes and the more anterior transmodal/heteromodal areas of prefrontal, temporal, and insular cortex [1, 2]. Building on this concept and borrowing from Sir Charles Sherrington’s original usage in 1906 [3], we adopt the terms: exogenous and endogenous brain systems. We propose that the “exogenous brain system” is a generalization of the sensorimotor pole of the SA axis that also encompasses the subcortical connections that are foundational to the registration of incoming visual, auditory, tactile, gustatory, olfactory, proprioceptive, and vestibular electro-chemical signals from inside and outside the corporeal body. In contradistinction, the “endogenous brain system” would then derive from the association pole of the SA axis and include the subcortical regions responsible for higher-order processing, or metacognition, including attention, salience, valence, reasoning, planning, action, language, emotion, social cognition, and impulse regulation. Since slower maturing endogenous networks require input from earlier developing exogenous networks for their own proper wiring and firing, sensory disorders in children offer a useful paradigm to study these developmental interactions.

Sensory over-responsivity (SOR) is a common presenting clinical symptom and is encompassed by the more expansive term: sensory processing disorder (SPD) [4]. Clinically, SOR is characterized as discomfort from everyday environmental stimuli that are typically considered non-noxious [5, 6]. Individuals with SOR often exhibit atypical and extreme externalizing behaviors (e.g., outbursts, meltdowns, or physical and verbal assaults to themselves and others) or internalizing behaviors (e.g., shutdowns, withdrawal, or avoidance) in response to innocuous stimuli such as seams in socks, vacuum cleaners, flashing lights, and specific food tastes or textures [7, 8]. SOR has garnered significant attention in neuroscience, occupational therapy, and educational milieus due to the recognition that approximately 70% [9, 10] of children with autism spectrum disorder (ASD), 46 to 69% [11, 12] of children with attention deficit hyperactivity disorder (ADHD), and 8 to 28% [13] of children without ascertained psychiatric diagnosis will experience clinically significant aversion to typically non-noxious stimuli. In community-based samples of school-aged children, the overall prevalence of SOR has been estimated to range from 2.8 to 6.5% [14, 15]. In a large cohort of more than 11,000 school-age children, those with SOR constituted 18% of the overall sample but 57% of children with clinically significant emotional dysregulation [16].

Anatomically, early sensory white matter (WM) pathways have been implicated in this condition in diffusion MRI (dMRI) studies focused on SPD and shown to correlate with sensory severity, somatization, and emotional disturbance [17–19]. In functional MRI (fMRI) studies of SOR in autistic individuals, SOR is characterized by hyperconnectivity, heightened neural activation, and reduced habituation in brain networks (salience) and sensory-limbic regions (primary sensory cortices, amygdala, orbitofrontal cortex, hippocampus) responsible for sensory detection, registration, valence attribution, information integration, and reward-related behavior [20–23]. Resting state fMRI (rs-fMRI) analysis of the Adolescent Brain Cognitive Development (ABCD) study data identified reduced functional connectivity (FC) within and between sensorimotor networks, enhanced FC between sensorimotor and salience networks, and altered FC between sensory networks and the bilateral hippocampi of parent-reported SOR in adolescents [16]. Altogether, these findings suggest microstructural white matter deficits and FC differences associated with SOR symptoms, signifying potential brain-based biomarkers for SOR [24, 25].

In this study, SOR is advanced as an understudied yet quintessential condition to investigate the neural connectivity of exogenous and endogenous systems when the basis of human functioning—sensory processing—falters. We incorporate multimodal neuroimaging, utilizing rs-fMRI alongside dMRI and structural MRI (sMRI), to investigate the effects of sensory processing challenges in neurodiverse (ND) school-age children. We specifically investigate two local FC measures, fractional amplitude of low-frequency fluctuations (fALFF) and regional homogeneity (ReHo), as well as long-range FC via dual regression (DR). In keeping with these prior findings from ASD as well as dMRI results from white matter in SOR, we postulate that in neurodiverse children, long-range FC will be reduced in those with SOR (ND/SOR) compared to those without (ND/NO-SOR). We further hypothesize that ND/SOR children will exhibit reduced local FC in exogenous networks but elevated local FC in endogenous networks. In a *post hoc* analysis, we hypothesize that a multimodal classification of SOR in a neurodiverse cohort that includes structural and functional neuroimaging metrics will outperform either alone. Finally, to probe the primary presenting parental concern in this cohort, emotional regulation [26], we subgroup the participants by behavioral regulation—categorizing children as “resilient” or “dysregulated”—and quantify exogenous and endogenous local FC in a data-driven behavioral and fMRI analysis.

## Methods

### Participants

Children (*n* = 190) in this study were serially recruited from an insurance-based community neurodevelopment center, Cortica Health Care (San Rafael, California). All neuroimaging was performed at the University of California, San Francisco (UCSF). Children were between 8 and 12 years of age at the time of behavioral and neuroimaging data collection using a study protocol approved by the UCSF institutional review board including written informed consent from the parent or caregiver and assent from the participant, with details previously reported [18]. Inclusion was based on a 12-item questionnaire, Early Symptomatic Syndromes Eliciting Neurodevelopmental Clinical Examinations-Questionnaire-Revised (ESSENCE-Q-REV) [27], designed to screen for a range of conditions, including ASD, ADHD, language impairments, and developmental coordination disorder. Children were included in the study if their caregiver reported at least one “yes” or two “maybe/a little” responses, indicating a high likelihood of meeting criteria for a neurodevelopmental condition. The ESSENCE-Q-REV has been used to screen children in the same age range as our study [6, 28, 29].

Exclusion criteria for this study included Autism (based on exceeding threshold scores on both the Social Communication Questionnaire [30] and the Autism Diagnostic Observation Schedule, Second Edition [31]), a nonverbal IQ of 70 or below, inability to complete assessment forms, exposure to in-utero toxins, gestational age of less than 32 weeks, intrauterine growth restriction (birth weight under 1500 g), significant hearing or vision impairments, active epilepsy, malignancy, or known or suspected brain injury/malformation. Medical concerns identified from the comprehensive neurodevelopmental assessment were further examined and resolved by the study’s pediatric neurologist (EJM) and pediatric neuroradiologist (PM).

### Cognitive, behavioral, and sensory assessments

All enrolled children in this study were assessed using the Wechsler Intelligence Scale for Children, Fifth Edition (WISC-V) [32], the Behavior Assessment System for Children, 3rd edition (BASC-3) [33], the Short Sensory Profile [34], and the Sensory Processing 3 Dimensions Assessment (SP3D:A) [35]. For details of the SP3D:A and its use to classify children into ND/SOR and ND/NO-SOR categories, please see Wren-Jarvis et al. (2024) [18].

### MRI data acquisition

Participants were scanned on a 64-channel head coil Siemens 3 Tesla (3T) Prisma MRI scanner (Erlangen, Germany). Structural T1-weighted (T1w) MPRAGE scans [TR = 2.3s, TE = 2.9ms, flip angle = 9º, voxel size = 1 mm isotropic] and fMRI scans [TR = 1.29s, TE = 32.4ms, flip angle = 45º, voxel size = 2.2 mm isotropic] were acquired. Additionally, two B0 field maps with reverse phase encoding directions (anterior–posterior (AP) and posterior–anterior (PA)) [TR = 1.85s, TE = 65.6ms, flip angle = 90º, voxel size = 2.2 mm isotropic] were acquired for B0 field distortion correction. The fMRI and field map scans utilized a single-shot echoplanar imaging sequence with multiband excitation, leveraging an acceleration factor of 4. The duration of an fMRI run totaled 6.02 min with 280 whole-brain volumes. A second identical fMRI acquisition was performed in those individuals who could tolerate the additional scan time. Further procedural details of the MR imaging protocol can be found in Wren-Jarvis et al. (2024) [18].

### Image quality assurance and preprocessing

Visual inspection of image quality was independently conducted by two raters (HC, JWJ) to identify T1 and fMRI scans with artifacts including excessive motion, ghosting, or ringing, for which no adequate preprocessing solutions currently exist. All visual inspection of MRI was performed blinded to any group membership and according to the order of participant scans. A scan was eliminated if only both raters concurred on the necessity of excluding it. Then, the first two volumes of each qualified fMRI scan were discarded, as commonly done, to allow the magnetic field to reach a steady-state equilibrium [36]. Only participants with both an approved T1 and fMRI scan were preprocessed by *fMRIPrep* 23.0.2 [37, 38], which is based on *Nipype* 1.8.6 [39, 40]. See supplementary text for details on the *fMRIPrep* processes involved in anatomical data, field map, and functional data preprocessing.

### fMRI processing and functional connectivity metrics

Following the application of *fMRIPrep*, functional scans exhibiting a mean framewise displacement (FD) >0.5 mm were excluded, as commonly done [41]. For participants with multiple functional runs meeting the quality criteria, a single run with the lowest FD was selected for primary analysis. Retest analyses utilized the next optimal scan. The *fMRIPrep* reports for each participant were reviewed to ensure accurate tissue segmentation, spatial normalization, susceptibility distortion correction, and registration between functional and anatomical data. Denoising involved the regression of several components: “aggressive” Independent Component Analysis-Automatic Removal of Motion Artifacts (ICA-AROMA) [42] noise regressors; average signals from white matter (WM) and cerebrospinal fluid (CSF); temporal derivatives, quadratic terms, and interaction terms of WM and CSF; and cosine regressors with a 128 s cutoff, all in a single step [42, 43]. Smallest Univalue Segment Assimilating Nucleus (SUSAN) [44] filtering, with a 6 mm full width at half maximum (FWHM) kernel, was applied for smoothing, chosen for its edge-preserving properties over traditional Gaussian smoothing.

Functional connectivity networks (FCNs) were generated based on a 17-component decomposition, using the Multivariate Exploratory Linear Optimized Decomposition into Independent Components (MELODIC) [45, 46] algorithm with multi-session temporal concatenation. For retest analysis, FCNs were generated based on an 18-component MELODIC decomposition. A group mask was provided to the MELODIC algorithm by temporally concatenating the preprocessed fMRI brain masks of all subjects, provided by *fMRIPrep*, followed by averaging these masks across time and applying a binarization threshold of 75%. The MELODIC algorithm facilitates the separation of mixed brain signals into spatial and associated temporal components that represent underlying neural connectivity patterns characteristic of FCNs, or alternatively, noise. Classification of FCNs was guided by visual inspection by our team neuroradiologist (PM).

Following the generation of FCNs, subject-specific long-range FC parameter maps were estimated for each FCN via Dual Regression (DR) [47, 48] in which the group-level ICA components from MELODIC were used as spatial regressors within a subject-specific general linear model (GLM) analysis to generate subject-specific time courses. Subsequently, these time courses were then employed as temporal regressors in a second GLM to produce subject-specific spatial maps. Each map can be interpreted as the expression of an FCN for that subject, showing how strongly each voxel participates in the specific FCN identified by group-level analysis.

In addition to DR, a measure of long-range FC, local FC parameters were also computed, including fractional Amplitude of Low-Frequency Fluctuations (fALFF) [49] and Regional Homogeneity (ReHo) [50], using the Configurable Pipeline for the Analysis of Connectomes (C-PAC) [51]. fALFF, a measure of local brain activity, involves bandpass filtering the fMRI data between 0.01 and 0.1 Hz (to focus on physiologically relevant low-frequency oscillations), calculating the standard deviation of both the filtered (ALFF) and unfiltered fMRI signals within a whole-brain mask, and then finally computing fALFF by dividing ALFF by the standard deviation of the unfiltered signal. The resulting fALFF maps provide a voxel-wise quantification of the proportion of low-frequency oscillations relative to the overall brain signal, thereby highlighting regions of increased or decreased local neuronal activity.

ReHo is another local voxel-based measure used to assess the similarity or synchronization of the time series of a given voxel with its neighboring voxels. The process for computing ReHo involves identifying the nearest neighbors for each voxel within a whole-brain mask, which leads to the formation of clusters comprising 7, 19, or 27 voxels within 3D space. Subsequently, Kendall’s coefficient of concordance (KCC) is computed for the time series of these clustered voxels for each voxel in the brain. The derived ReHo map provides a spatial representation of local synchronization, where higher ReHo values indicate areas of elevated local FC or homogeneity. 

Altogether, the incorporation of Dual Regression, fALFF, and ReHo, as measures of FC in our study, is in line with modern standard protocol for rs-fMRI analysis [52, 53] and allows for comparison of long-range versus local FC, generalization of findings across measures, and investigation of FC at different levels of spatial granularity (e.g., whole-brain, networks, lobes, regions). In general, all rs-fMRI analysis methods that are employed in this study have been previously developed and made ready for use through open-source software.

### Functional connectivity network, whole-brain gray matter, lobe, cortical region, and subcortical region masks

Here, we present the levels of spatial granularity we examine and detail the masking procedure for each level. We first present functional connectivity networks, which are generated by data-driven methods. Then, we present the whole-brain and its constituent lobes and regions, derived from a standard atlas.

#### Functional connectivity network (FCN) masks

Voxel-wise, Z-score normalization (*Mean* = 0, *SD* = 1) was consistently applied to each subject’s FC map of the three key metrics: DR, fALFF, and ReHo. To conduct a network-wise analysis of each metric, FCN masks were generated by binarizing (1 if *x* > 0; 0 otherwise) the already thresholded Z-statistic images corresponding to each FCN, which are provided in the MELODIC outputs. Utilizing these FCN masks enabled the calculation of average values within each FCN mask for each subject, with each FCN-specific DR parameter map constrained by its corresponding FCN mask. Furthermore, the same FCN masks were applied to mask each subject’s fALFF and ReHo images, facilitating the calculation of mean values of the masked voxels for each subject, metric, and FCN. Finally, before statistical analysis, standardization was applied per FCN to account for differences in FC range.

#### Whole-brain gray matter (GM) mask

A global gray matter (GM) mask was also defined for broader analysis (Supplementary Fig. 1A). This was achieved by employing FSL’s FAST (FMRIB’s Automated Segmentation Tool) [54] to segment the gray matter of the T1w *MNIPediatricAsym: cohort-4* standard space template with 2 mm isotropic resolution, tailored for subjects aged 7.5–13.5 (spanning from pre to mid-puberty) [55]. This segmentation allowed for the calculation of mean fALFF and ReHo values within the GM voxels, adopting a similar approach to that used for the FCN masks. However, it is important to reiterate that dual regression is inherently linked to individualized FCN parameter maps, and therefore, only singular image metrics containing whole-brain GM in its entirety, such as fALFF and ReHo, are applicable in this context.

#### Lobe, cortical region, and subcortical region masks

FreeSurfer [56] 7.4.0’s recon-all pipeline [57] integrated with SynthSeg [58] was run on the *MNIPediatricAsym: cohort-4* template in order to define standardized brain regions based on the Desikan-Killiany-Tourville (DKT) atlas [59]. Based on the segmented and parcellated template, masks of cortical and subcortical regions, separate for the left and right hemispheres, were generated. 39 regions in each hemisphere were delineated for a total of 78 regions (Supplementary Fig. 2). 8 lobe masks (Supplementary Fig. 3) were generated by grouping regions according to the appendix of Klein and Tourville (2012) [59]. Again, this analysis only pertains to fALFF and ReHo.

### Structural connectivity

In addition to measures of FC, we examine the brain macrostructure and microstructure of our population, readily available due to our multimodal scanning protocol and prior research, to examine their associations with our FC measures and findings. Below, we discuss our macrostructural metrics followed by our microstructural metrics.

#### Brain morphometry and macrostructure

In addition to functional imaging, measures of brain morphometry and macrostructure: volume, surface area, and cortical thickness were examined between ND/SOR and ND/NO-SOR groups. These metrics were readily provided from the run of FreeSurfer 7.3.2’s recon-all [57] during preprocessing through *fMRIPrep*. Cortical and subcortical regions were defined by the DKT atlas. Lobes were defined according to the appendix of Klein and Tourville (2012) [59]. A full list of regions can be seen in Supplementary Fig. 2.

#### Diffusion tensor imaging and neurite orientation dispersion and density imaging

Diffusion tensor imaging (DTI) metrics of fractional anisotropy (FA), axial diffusivity (AD), mean diffusivity (MD), and radial diffusivity (RD) as well as neurite orientation dispersion and density imaging (NODDI) metrics of neurite density index (NDI), orientation dispersion index (ODI), and isotropic volume fraction (FISO) were examined between ND/SOR and ND/NO-SOR groups based on preprocessing from Wren-Jarvis et al. (2024) [18]. A total of 48 tracts were tested, including 21 tracts with homologous pairs and 6 tracts without. For a full list of WM tracts, please see Table 1 of Mark et al. (2023) [60].

### Statistical analyses

Our statistical methods are presented in the order they are to appear in the Results section. Again, all statistical methods follow modern standard protocol for rs-fMRI analysis [52, 53, 61]. Methods specific to rs-fMRI are permutation-based voxel-wise and seed-based connectivity analyses. The other statistical methods are more typical and widely accepted.

#### PCA and correlation matrices

Principal component analysis (PCA) was employed using the ‘scikit-learn’ library [62] to elucidate the behavior of FCNs across all FC metrics as well as lobes and regions for fALFF. Subsequently, correlation matrices based on FCNs were computed for each FC metric. Correlations were calculated using the Pearson correlation coefficient (*r*) from the ‘pandas’ library [63] and subjected to Benjamini-Hochberg (BH) false discovery rate (FDR) corrections for multiple comparisons using the ‘statsmodels’ library [64]. PCA and correlational analyses were implemented in Python 3.9.6 [65].

#### Permutation-based voxel-wise analysis

Permutation-based voxel-wise analysis using ‘randomise’ [66] paired with threshold-free cluster enhancement (TFCE) [67] from FSL was used to examine FC between ND/SOR and ND/NO-SOR groups. For dual regression, analyses were performed on the ICA-generated FCNs, and for fALFF, ReHo, and seed-based connectivity, analyses were performed on whole-brain maps. Two-sided two-sample t-tests were conducted with test one investigating ND/SOR >ND/NO-SOR and test two investigating ND/SOR < ND/NO-SOR. TFCE corrects for multiple comparisons across the FCN or whole brain. We defined significance at *p* < 0.025 to account for two-sided tests.

#### Interactions between SOR status and network type

Two-way mixed ANOVAs for each FC metric: dual regression, fALFF, and ReHo were performed, employing a 2 × 2 factorial design. The between-subjects factor was *SOR status* (ND/SOR, ND/NO-SOR), and the within-subjects factor was *network type* (exogenous, endogenous). Please see the section on handling ANOVAs for details on meeting assumptions.

#### ND/SOR versus ND/NO-SOR group differences: whole-brain GM, FCNs, lobes, cortical and subcortical regions, and WM tracts

Group differences (ND/SOR vs. ND/NO-SOR) were examined over FC metrics for whole-brain GM, FCNs, lobes, and cortical and subcortical regions; over macrostructural metrics for cortical and subcortical regions; and over diffusion metrics for WM tracts using the Mann-Whitney U test (‘scipy.stats’ library [68]) with BH corrections. Descriptive statistics including mean, standard deviation, and Cohen’s *d* effect size using the ‘pingouin’ library [69] were implemented in Python 3.9.6.

#### Classifying ND/SOR versus ND/NO-SOR

We aimed to distinguish between ND/SOR and ND/NO-SOR individuals using fALFF. The fALFF data was standardized to ensure each FC feature (network, lateralized region) had a mean of zero and a standard deviation of one. Feature selection was conducted using the ‘SelectKBest’ method, employing ‘f_classif’ as the selection criterion. This method prioritizes features that are most predictive of the outcome by their F-statistics. To evaluate features and model performance, repeated stratified k-fold cross-validation, with a configuration of 5 folds and 25 repetitions was employed, totaling 125 runs. Feature importance was evaluated by setting K features as equal to the number of available features so that each feature is chosen and provided an F-score as well as p-value 125 times. We performed 3 rounds of testing, round 1 with only FCN fALFF, round 2 with only region fALFF divided by hemisphere (i.e., lateralized regions), and round 3 with lateralized regions involving FC (fALFF) and morphometry (volume, surface area, cortical thickness) as well as lateralized tracts involving diffusion metrics (DTI: FA, AD, MD, RD and NODDI: NDI, ODI, FISO). When using FCNs for ND/SOR vs. ND/NO-SOR classification, K = 16 (14 FCNs, age, and sex). When using lateralized regions, K = 80 (78 regions, age, and sex). When using all metrics, K = 660 (658 brain features, age, and sex).

Prior to applying classification algorithms for hypothesis testing, we selected the top features for each round of testing. We include all significant features per round if there are less than 15, else we include the top 15 significant features. Then, we run all combinations of groups of two or more of the top features through PCA to acquire the first principal component (PC1) and subsequently evaluate every PC1 obtained through machine learning algorithms. A suite of classification algorithms was implemented: gaussian naïve bayes (GNB), k-nearest neighbors (KNN), linear discriminant analysis (LDA), logistic regression, support vector machine (SVM) with linear and radial basis function (RBF) kernels, and random forest. Model assessment was based on the area under the curve for the receiver operating characteristic (AUC-ROC). We report the classifier and brain structures of the first principal component that result in the optimal AUC-ROC along with standard error. All classification preprocessing and analyses are conducted in Python 3.9.6 using the ‘scikit-learn’ module [62].

#### Seed-based connectivity analyses

Seed-based connectivity analyses (SBCA) were conducted post hoc to examine long-range functional connectivity of regions of interest (ROIs) emphasized following FC analyses. Permutation-based voxel-wise analyses were conducted on seed-based connectivity (SBC) brain maps which were constructed by correlating the mean time series of individual ROIs with the timeseries of all other voxels within a whole-brain mask. For region-to-region SBCA, the mean timeseries of the voxels from a particular region (start node) was used to correlate with the timeseries of all other voxels within a subject’s masked end node. Then, the mean of the correlations within the masked end node was computed to achieve a measure of SBC to the start node.

#### Structural covariance

Similar to SBCA, structural covariance was examined post hoc between ROIs identified following FC analyses. Correlations between the cortical thicknesses of ROIs were examined to compare ND/SOR and ND/NO-SOR groups. Correlations were conducted using the Pearson correlation coefficient (*r*) from the ‘pandas’ library [63].

#### Resilient versus dysregulated behavior

The Behavioral Assessment Scale for Children, 3rd edition (BASC-3) is utilized for behavioral analysis. The features of the assessment are standardized prior to applying latent profile analysis (LPA) through the ‘tidyLPA’ library [70] in R [71]. The behavioral characteristics (BASC-3 scales and indices) of the profiles are analyzed using Mann-Whitney U tests with BH corrected p-values and Cohen’s *d*. FC metrics (fALFF, ReHo) are examined in the context of the behavioral profiles using a three-way mixed ANOVA with a 2 × 2 × 2 factorial design, which includes *SOR status* (ND/SOR, ND/NO-SOR) and *behavior* (dysregulated, resilient) as between-subjects factors and *network type* (exogenous, endogenous) as a within-subjects factor.

#### Handling ANOVAs

Prior to conducting the ANOVAs, we tested for normality using Shapiro-Wilk tests and confirmed with Q-Q plots [72]. We also tested for homogeneity of variance using Levene’s test and homogeneity of covariances using Box’s M-test. All tests were conducted using the ‘rstatix’ library [73] in R [71]. ANOVAs were also conducted using R with the ‘ez’ library [74], which checks for the assumption of sphericity using Mauchly’s test and reports Greenhouse-Geisser corrected p-values when tests for sphericity are significant. No Greenhouse-Geisser corrected p-values were reported for our analysis, indicating that the assumptions for sphericity were met. We report the F statistics and generalized eta squared ($\:\eta\:$_*G*_^*2*^). Post hoc Mann-Whitney U tests were conducted to examine significant main effects and interactions.

#### Handling p-values

All reported p-values are rounded to three decimal places.

## Results

### Demographics

Participants (*n* = 123) recruited from community neurodevelopment clinics underwent an MRI scan on a Siemens 3 T Prisma scanner using multiband echoplanar imaging at 2.2-mm spatial resolution, including a 6-minute resting state fMRI sequence while viewing a fixation cross and a second identical sequence in cooperative participants. The children also underwent multi-shell multiband dMRI with scanning parameters detailed in Wren-Jarvis et al. (2024) [18]. Twenty-three participants were excluded based on visual inspection of the MRI data, and 17 additional participants were excluded due to an fMRI mean framewise displacement (FD) greater than 0.5 mm, resulting in a final sample of 83 participants [*Mean age*: 10.47 ± 1.59 years; 30 females]. Participants were categorized as ND/SOR (*n* = 39) or ND/NO-SOR (*n* = 44) based on whether they exhibited auditory, tactile, or visual SOR utilizing the Sensory Processing 3 Dimensions Assessment (SP3D:A). The SP3D:A is a direct assessment tool administered by licensed pediatric occupational therapists (RC, LR, RG, and KT) observing the child’s behavior in response to structured, reproducible stimuli across several sensory modalities [18, 35]. Prior work has validated the intrinsic existence of ND/SOR and ND/NO-SOR subtypes through a data-driven analysis of the SP3D:I, or inventory, a related parent-report measure of sensory processing [26]. Table 1 provides sensory, cognitive, behavioral, and demographic information. With the exception of SOR, no significant differences were observed between ND/SOR and ND/NO-SOR cohorts with regard to risk for neurodevelopmental disorder conditions as assessed by the ESSENCE-Q-REV [27], communication skills and social functioning as indicated by the Social Communication Questionnaire [30], general sensory symptomology measured by the Short Sensory Profile [34], intellectual capacity as assessed using the Wechsler Intelligence Scale for Children, Fifth Edition (WISC-V) [32], or common co-occurring clinical conditions determined with the Behavioral Assessment Scale for Children, 3rd edition (BASC-3) [33]. Both groups displayed low levels of subject motion. However, the ND/SOR [*Mean* = 0.184, *SD* = 0.063] group showed significantly lower FD [*U* = 601, *p* = 0.019, *d* = −0.65] than the ND/NO-SOR [*Mean* = 0.239, *SD* = 0.010] group.

**Table 1:** Participant phenotypic and demographic information

Set	Evaluation	Index	ND/SOR (*n* = 39)	ND/NO-SOR (*n* = 44)	Mann-Whitney U	*p*-value	Cohen’s d
Test (*n* = 83)	**Age**	**Year** (µ ± 𝜎)	10.62 ± 1.56	10.34 ± 1.63	937.5	0.471	0.17
	**ESSENCE-Q-REV**	**Total Score** (µ ± 𝜎)	11.15 ± 4.48	11.19 ± 4.71	829	0.933	−0.01
	**SCQ**	**Total Score** (µ ± 𝜎)	7.21 ± 5.59	8.20 ± 5.84	770	0.423	−0.17
	**SSP**	**Total Score** (µ ± 𝜎)	138.26 ± 20.82	136.00 ± 20.34	884.5	0.812	0.11
	**SP3D:A**	**Over-Responsivity Total Score** (µ ± 𝜎)	12.28 ± 1.81	10.00 ± 0.00	1716	< 0.001***	**1.85**
	**WISC-V**	**VCI** (µ ± 𝜎)	111.59 ± 15.83	110.93 ± 13.01	847	0.923	0.05
		**VSI** (µ ± 𝜎)	107.26 ± 13.95	111.05 ± 13.80	754.5	0.346	−0.27
		**FRI** (µ ± 𝜎)	110.23 ± 14.47	106.23 ± 13.59	1003	0.186	0.29
		**WMI** (µ ± 𝜎)	100.23 ± 16.13	101.98 ± 13.70	812	0.677	−0.12
		**PSI** (µ ± 𝜎)	92.33 ± 16.09	96.02 ± 13.29	757	0.358	−0.25
	**BASC-3** **(n = 71)** ND/SOR (*n* = 31) ND/NO-SOR (*n* = 40)	**Anxiety** (µ ± 𝜎)	17.74 ± 7.97	16.73 ± 6.48	673	0.542	0.14
		**Depression** (µ ± 𝜎)	11.87 ± 5.93	10.80 ± 6.20	701	0.350	0.18
		**ADHD PI** (µ ± 𝜎)	17.45 ± 5.24	17.33 ± 4.70	622.5	0.981	0.03
		**Autism PI** (µ ± 𝜎)	13.84 ± 8.63	14.13 ± 9.25	620	1.000	−0.03
**Set**	**Evaluation**	**Index**	**ND/SOR (n = 32)**	**ND/NO-SOR (n = 22)**	**Mann-Whitney*U***	**p-value**	**Cohen’s*d***
Retest (*n* = 54)	**Age**	**Year** (µ ± 𝜎)	10.72 ± 1.57	10.44 ± 1.68	379	0.641	0.17
	**ESSENCE-Q-REV**	**Total Score** (µ ± 𝜎)	10.97 ± 4.01	11.05 ± 4.91	334.5	0.985	−0.02
	**SCQ**	**Total Score** (µ ± 𝜎)	6.91 ± 4.66	6.91 ± 5.12	360	0.895	−0.00
	**SSP**	**Total Score** (µ ± 𝜎)	141.94 ± 18.27	145.00 ± 15.49	315.5	0.526	−0.18
	**SP3D:A**	**Over-Responsivity Total Score** (µ ± 𝜎)	12.44 ± 1.93	10.00 ± 0.00	704	< 0.001***	**1.63**
	**WISC-V**	**VCI** (µ ± 𝜎)	110.88 ± 17.01	111.00 ± 15.11	334.5	0.764	−0.01
		**VSI** (µ ± 𝜎)	105.94 ± 14.56	112.64 ± 13.73	264	0.123	−0.47
		**FRI** (µ ± 𝜎)	109.31 ± 15.66	109.73 ± 14.56	330	0.704	−0.03
		**WMI** (µ ± 𝜎)	99.97 ± 16.18	102.41 ± 13.33	317	0.542	−0.16
		**PSI** (µ ± 𝜎)	92.53 ± 16.38	94.32 ± 14.48	354.5	0.972	−0.11
	**BASC-3** **(n = 47)** ND/SOR (*n* = 26) ND/NO-SOR (*n* = 21)	**Anxiety** (µ ± 𝜎)	18.00 ± 8.51	13.43 ± 4.19	364.5	0.051	0.66
		**Depression** (µ ± 𝜎)	12.38 ± 6.31	10.10 ± 5.20	325.5	0.265	0.39
		**ADHD PI** (µ ± 𝜎)	17.42 ± 5.15	17.38 ± 4.93	267	0.906	0.01
		**Autism PI** (µ ± 𝜎)	14.35 ± 8.35	12.19 ± 8.87	332.5	0.206	0.25

Summary statistics—including the mean (µ) and standard deviation (𝜎) for each group (ND/SOR, ND/NO-SOR), p-value following Mann-Whitney U tests, and effect size indicated by Cohen’s *d*—are reported for test and retest sets

*ESSENCE-Q-REV* ESSENCE-Q Revised, *SCQ* Social Communication Questionnaire, *SSP* Short Sensory Profile, *SP3D:A* Sensory Processing 3 Dimensions Assessment, *WISC-V* Wechsler Intelligence Scale for Children, Fifth Edition, *VCI* Verbal Comprehension Index, *VSI* Visual Spatial Index, *FRI* Fluid Reasoning Index, *WMI* Working Memory Index, *PSI* Processing Speed Index, *BASC-3* Behavior Assessment System for Children, 3rd edition, *ADHD* Attention Deficit Hyperactivity Disorder, *PI* Probability Index, *ND/SOR* Neurodiverse children with sensory over-responsivity, *ND/NO-SOR* Neurodiverse children without sensory over-responsivity

Of the 83 participants, 54 [*Mean age*: 10.61 ± 1.61 years; 17 females; 32 SOR] had a second fMRI acquisition that met quality criteria for test-retest analysis. Similarly, no significant differences apart from SOR were observed between retest ND/SOR and ND/NO-SOR subgroups across phenotypic assessments (Table 1). The ND/SOR [*Mean* = 0.225, *SD* = 0.098] and ND/NO-SOR [*Mean* = 0.253, *SD* = 0.094] subgroups showed no significant differences in FD [*U* = 286, *p* = 0.249, *d* = −0.29].

### Functional connectivity measures delineate SOR in neurodiverse children

#### Functional connectivity: exogenous and endogenous brain systems defined by canonical networks

**Fig. 1:**
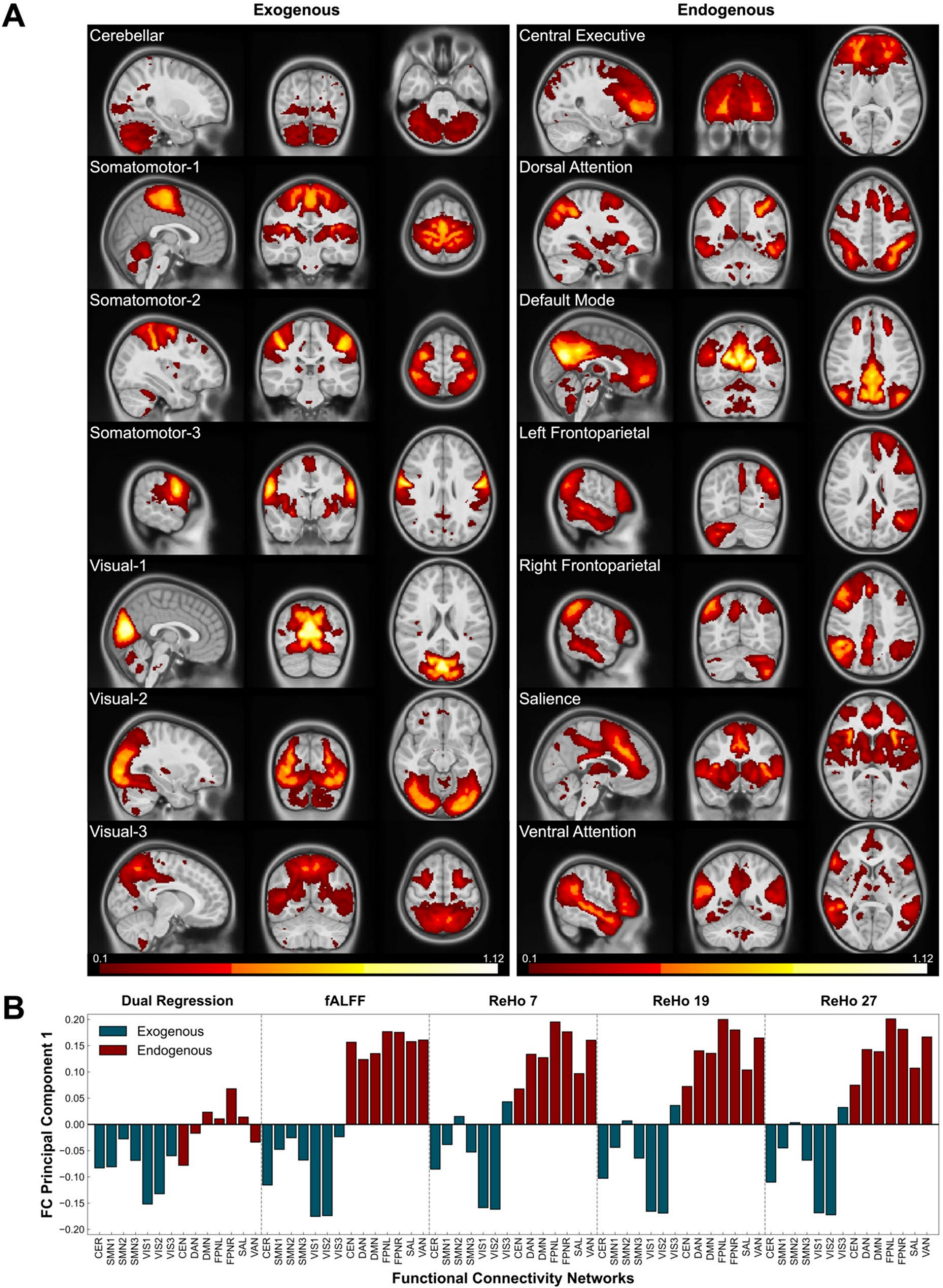
Data driven determination of exogenous and endogenous brain systems from canonical functional connectivity networks. (**A**) Resting state functional MRI data for all participants generated 14 stable functional connectivity networks based on MELODIC probability estimates for each voxel’s association with specific independent components. These networks have been subjected to thresholding, Gaussian smoothing with a full width half maximum (FWHM) kernel of 4 mm for the purpose of visualization, and are displayed on top of the *MNIPediatricAsym: cohort-4* standard template. They are grouped by exogenous (left) and endogenous (right) brain systems. (**B**) First principal component loading, denoted on the y-axis, across long-range and local functional connectivity metrics explains 27.4% of the variance. FC, functional connectivity; MELODIC, multivariate exploratory linear optimized decomposition into independent components; fALFF, fractional amplitude of low-frequency fluctuations; ReHo, regional homogeneity

Following data-driven MELODIC decomposition of the preprocessed rs-fMRI data, 14 stable functional connectivity networks (FCNs) were identified in both test and retest samples; 3 noise components were identified in the test sample and 4 were identified in the retest sample (Fig. 1A, Supplementary Fig. 4A). Exogenous and endogenous network clusters were determined by local connectivity differences, captured by a separation between network types in the first principal component (PC1) (Fig. 1B, Supplementary Fig. 4B). The exogenous networks (Fig. 1A, Supplementary Fig. 4A), which consist of the visuo-cerebellar (CER), somatomotor-1 (SMN1), somatomotor-2 (SMN2), somatomotor-3 (SMN3), visual-1 (VIS1), visual-2 (VIS2), and visual-3 (VIS3) networks, are primarily influenced by photo-electrical and mechanical inputs from the environment (e.g., tactile, visual) or body (e.g., proprioceptive, vestibular). The unsupervised decomposition subdivided the somatomotor and visual systems into three distinct networks each. The visual networks were arranged into primary, secondary, and tertiary networks extending medially to laterally, mirroring the hierarchical organization of the visual cortex. The somatomotor networks were organized topographically, representing the somatosensory and motor homunculi. The endogenous networks (Fig. 1A, Supplementary Fig. 4A), including the central executive (CEN), dorsal attention (DAN), default mode (DMN), left frontoparietal (FPNL), right frontoparietal (FPNR), salience (SAL), and ventral attention (VAN) networks, are responsible for higher-order integration processes and cognitive control, including decision-making, regulation, attention, emotion, memory, self-reflection, and mind wandering [75].

Similar FC network patterns were observed across ND/SOR and ND/NO-SOR groups for long-range (DR) and local (fALFF, ReHo) FC in the all-participant test sample (Fig. 1B) and recapitulated in the retest dataset (Supplementary Fig. 4B). Long-range FC, regardless of affinity to exogenous or endogenous systems, was positively correlated between all networks (Supplementary Fig. 5). However, in local FC analysis (fALFF, ReHo), the exogenous and endogenous networks were anti-correlated while the FCNs remained positively correlated within each endogenous and exogenous network type, indicating differences between long-range and local connectivity in all participants.

##### Long-range functional connectivity: dual regression (DR)

**Fig. 2:**
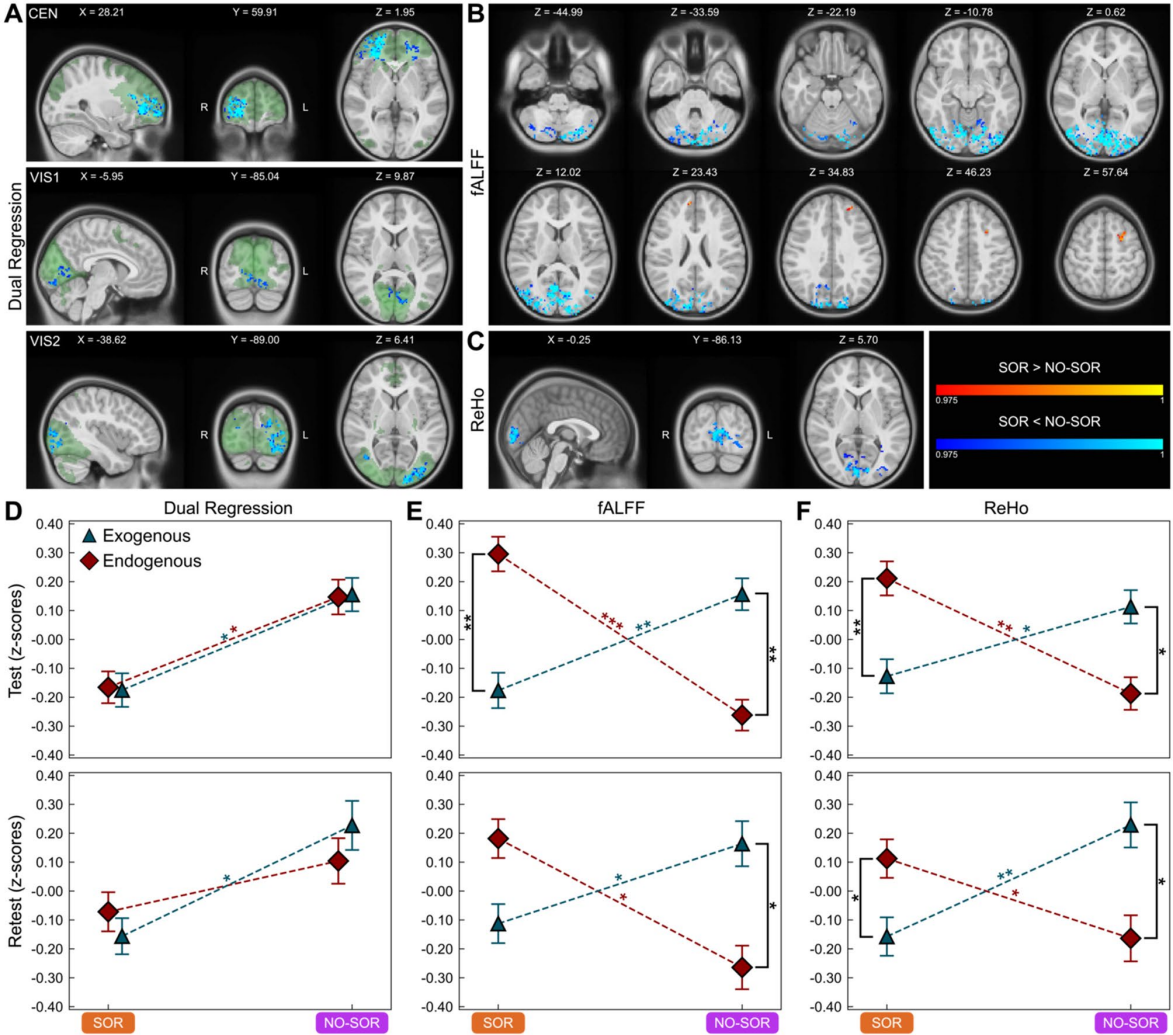
Sensory over-responsive children show decreased long-range and local exogenous but increased local endogenous functional connectivity. (**A**) Permutation-based voxel-wise analyses within single functional connectivity networks show decreased long-range connectivity specific to SOR status. FCNs displayed in green. (**B**) Permutation-based voxel-wise analysis based on fALFF and (**C**) ReHo 19 whole-brain maps demonstrates decreased local connectivity in posterior brain regions with increased connectivity in the left caudal middle frontal and bilateral superior frontal gyri (*p* ≤ 0.025). World coordinates based on *MNIPediatricAsym: cohort-4* standard template. (**D**) Long-range comparison reveals lower connectivity for the ND/SOR cohort in exogenous and endogenous brain systems for test (top row) and retest (bottom row) datasets; (**E** and **F**) local connectivity shows double dissociations where ND/SOR children have decreased exogenous and increased endogenous connectivity while ND/NO-SOR children show the reverse. Error bars represent standard error. Post hoc Mann-Whitney U test statistical significance indicated by: * *p* ≤ 0.05, ** *p* ≤ 0.01, *** *p* ≤ 0.001. CEN, central executive network; VIS1, primary visual network; VIS2, secondary visual network; fALFF, fractional amplitude of low-frequency fluctuations; ReHo, regional homogeneity; SOR and ND/SOR, neurodiverse children with sensory over-responsivity; NO-SOR and ND/NO-SOR, neurodiverse children without sensory over-responsivity; FCN, functional connectivity network

Data-driven, permutation-based, voxel-wise analysis within each FCN revealed significant clusters of DR differences at *p* ≤ 0.025 in the CEN (*n voxels* = 2,290), VIS1 (*n voxels* = 420), and VIS2 (*n voxels* = 1,083) networks when comparing neurodiverse children with and without SOR. Specifically, ND children with SOR displayed lower long-range connectivity in the right frontal and left occipital areas (Fig. 2A). Following a two-way mixed ANOVA with *SOR status* (ND/SOR, ND/NO-SOR) and *network type* (exogenous, endogenous) factors, there was a significant DR main effect for *SOR status* [*F*_(1,81)_ = 6.699, *p* = 0.011, η_*G*_^2^ = 0.061] but not *network type* (Supplementary Table 1). The retest sample confirms trends for this main effect [*F*_(1,52)_ = 2.539, *p* = 0.117, η_*G*_^2^ = 0.039]. Of note, the ND/SOR cohort demonstrated lower DR mean values in exogenous and endogenous network types (Fig. 2D, Supplementary Table 2) and individual FCNs (Supplementary Fig. 6, Supplementary Table 3) relative to the ND/NO-SOR cohort. Specifically, the ND/SOR group had significantly reduced exogenous long-range FC in VIS1 [*U* = 538, *p* = 0.022, *d* = −0.64] and VIS2 [*U* = 547, *p* = 0.022, *d* = −0.62] with similarly decreased endogenous long-range FC in CEN [*U* = 498, *p* = 0.015, *d* = −0.77] and SAL [*U* = 584, *p* = 0.044, *d* = −0.55] after adjusting for multiple comparisons.

##### Local functional connectivity: fractional amplitude of low-frequency fluctuations (fALFF)

Similar to the DR analysis, voxel-wise, whole-brain differences showed significantly (*p* ≤ 0.025) lower fALFF for ND/SOR children in the cerebellum and bilateral occipital areas of the brain (*n voxels* = 8,700), encompassing CER, VIS1, and VIS2. By contrast, the ND/SOR cohort shows increases in the medial frontal areas of the brain (*n voxels* = 133), encompassing the left caudal middle frontal and bilateral superior frontal gyri (Fig. 2B). Following a two-way mixed ANOVA with consistent factors of *SOR status* (ND/SOR, ND/NO-SOR) and *network type* (exogenous, endogenous), there was a significant interaction between the factors (Supplementary Table 1). This significant interaction between *SOR status* and *network type* holds for both test [*F*_(1,81)_ = 12.993, *p* = 0.001, η_*G*_^2^ = 0.101] and retest [*F*_(1,52)_ = 6.080, *p* = 0.017, η_*G*_^2^ = 0.071] datasets. In general, the ND/SOR cohort has lower exogenous network local FC (Fig. 2E, Supplementary Table 2). Notably, CER, VIS1, and VIS2 maintained significance after correction for multiple comparisons with CER [*U* = 420, *p* = 0.001, *d* = −1.00] and VIS2 [*U* = 440, *p* = 0.001, *d* = −0.88] displaying particularly strong effects. The SMN1, SMN2, SMN3, and VIS3 show no differences (Supplementary Fig. 6, Supplementary Table 3).

Conversely, the ND/SOR children exhibited stronger endogenous network local FC, evident in the CEN, DAN, FPNL, SAL, and VAN. Statistical significance was maintained after multiple comparisons corrections. The DMN and FPNR showed trend level differences in the same direction. The SAL [*U* = 1287, *p* = 0.001, *d* = 0.88] and VAN [*U* = 1246, *p* = 0.001, *d* = 0.70] networks exhibited notably strong fALFF differences (Supplementary Fig. 6, Supplementary Table 3). Therefore, the significant interaction was produced by ND children with SOR manifesting a substantial decrease of exogenous local FC paired with an increase in endogenous local FC, whereas the children without SOR displayed the opposite—an increase of exogenous local FC and decrease of endogenous local FC—resulting in a double dissociation across participant groups and network types.

##### Local functional connectivity: regional homogeneity (ReHo)

Similar to fALFF, voxel-wise, whole-brain differences (*n voxels* = 1,112) revealed significant increases in ReHo for ND/NO-SOR youth in the exogenous, occipital area of the brain, encompassing mainly VIS1 (Fig. 2C). When examining FCNs, all three ReHo cluster sizes (7, 19, 27) were consistent in their results (Fig. 1B, Supplementary Fig. 4B). However, for endogenous FCNs, effect size strengthened as cluster size decreased, while exogenous FCNs showed strengthened effect size with increased cluster size. Thus, ReHo with the intermediate cluster size of 19 was chosen for all the following analyses.

The ReHo two-way mixed ANOVA with *SOR status* and *network type* factors recapitulated the double dissociation pattern seen with fALFF for both the test and retest participant cohorts (Supplementary Table 1), [test: *F*_(1,81)_ = 8.400, *p* = 0.005, η_*G*_^2^ = 0.064; retest: *F*_(1,52)_ = 5.657, *p* = 0.021, η_*G*_^2^ = 0.067]. Again, ND children with SOR show significantly weaker exogenous local FC but significantly stronger endogenous local FC (Fig. 2F, Supplementary Table 2). When examining individual FCNs, two exogenous networks: VIS1 [*U* = 552, *p* = 0.025, *d* = −0.62] and VIS2 [*U* = 529, *p* = 0.019, *d* = −0.70] demonstrated the most diminished FC, whereas three endogenous networks: DAN [*U* = 1148, *p* = 0.029, *d* = 0.59], FPNL [*U* = 1236, *p* = 0.008, *d* = 0.78], and FPNR [*U* = 1129, *p* = 0.038, *d* = 0.49] exhibited the greatest heightened local FC for the ND/SOR children (Supplementary Fig. 6, Supplementary Table 3).

##### Local functional connectivity: lobes and regions

Using lobe (Supplementary Fig. 3) and region masks based on the DKT atlas [59], lobe and regional fALFF FC analysis revealed findings consistent with the FCN analysis. Following adjustment for multiple comparisons, the ND/SOR children exhibited weaker FC in the occipital lobe [*U* = 481, *p* = 0.002, *d* = −0.84] and cerebellum [*U* = 513, *p* = 0.004, *d* = −0.76] with stronger FC in the frontal lobe [*U* = 1241, *p* = 0.002, *d* = 0.80] (Supplementary Table 4). The more parcellated analysis, including 78 regions (39 per hemisphere), showed an exogenous versus endogenous divide. Regions that overlap with exogenous brain systems were reduced while the endogenous regions (i.e., medial anterior) were elevated (Supplementary Fig. 2, Supplementary Table 5). Bilaterally, the lateral occipital, pericalcarine, cerebellar cortex, and lingual areas—all belonging to exogenous FCNs—showed significant fALFF reduction for the ND/SOR children, whereas the pars opercularis (pOPER), caudal anterior cingulate (cACC), amygdala, caudate, and superior frontal regions, which all belong to endogenous FCNs, were significantly elevated. Supplementary Table 5 contains the full list of regions showing bilateral or unilateral differences between the ND/SOR and ND/NO-SOR cohorts following corrections for multiple comparisons.

##### Local functional connectivity: whole-brain gray matter

Whole-brain GM FC analysis was conducted to determine whether ND children with and without SOR showed differences at the cortical level in general. The analysis, based on a whole-brain GM segmentation mask (Supplementary Fig. 1A), revealed no significant differences between the two cohorts across local gray matter FC metrics in the test (Supplementary Fig. 1B, Supplementary Table 6) or retest datasets (Supplementary Fig. 1C, Supplementary Table 6). This prompted deeper examination of the potential macrostructural and microstructural differences and contributions to a sensory-based cohort classification.

#### Multimodal feature importance and classification of SOR in a neurodiverse pediatric cohort

Macrostructural analysis of gray matter volume, surface area, and cortical thickness was conducted using 3D T1-weighted images at 1-mm resolution. Following correction for multiple comparisons, there were no significant differences between the ND/SOR and ND/NO-SOR groups. Microstructural analysis of white matter integrity was based on the Johns Hopkins University Atlas for white matter (WM) tracts [76] (48 total) using tract-based spatial statistics [77] analysis of multi-shell, multiband dMRI data. Axial diffusivity (AD), a diffusion tensor imaging (DTI) [78] measure of water diffusion parallel to fiber tracts, is significantly lower for the ND/SOR group in the right posterior thalamic radiation (PTR) and retrolenticular limb of the internal capsule (RLIC) [rh.PTR: *U* = 440, *p* = 0.007, *d* = −0.93; rh.RLIC: *U* = 483, *p* = 0.015, *d* = −0.85]. Similarly, the isotropic volume fraction (FISO), a neurite orientation dispersion and density imaging (NODDI) [79] measure of extracellular free water movement, is significantly lower for the ND/SOR children in the left fornix/stria terminalis (FX/ST), left PTR, and right RLIC [lh.FX/ST: *U* = 485, *p* = 0.032, *d* = −0.79; lh.PTR: *U* = 529, *p* = 0.049, *d* = −0.71; rh.RLIC: *U* = 533, *p* = 0.049, *d* = −0.62] (Supplementary Table 7).

**Fig. 3:**
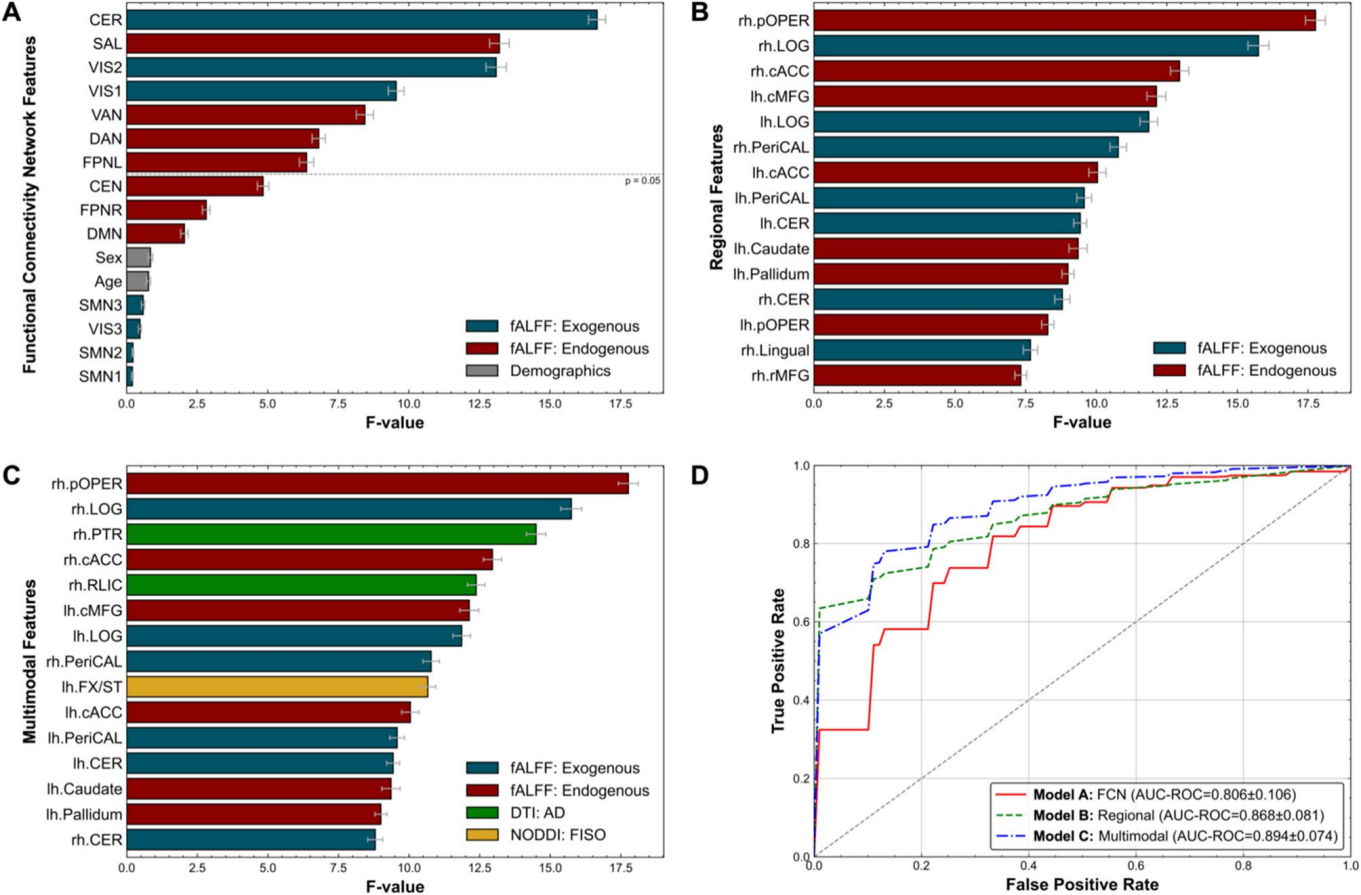
Multimodal neuroimaging features best classify sensory over-responsivity. (**A** to **C**) Feature selection based on F-statistic ranking for SOR discrimination was conducted for three datasets: (**A**) functional connectivity networks; (**B**) regional functional connectivity (FC) split by hemisphere; and (**C**) lateralized regional FC, regional morphometry metrics, and diffusion metrics of white matter tracts. Age and sex were also included as features. Only features significant at *p* ≤ 0.05 and within the top 15 rankings were evaluated by models. (**D**) Random forest classifier along with PC1 from the optimal combination of multimodal features had the highest AUC-ROC (Model C). The no-discrimination line is indicated in gray dashes. Performance is shown in parentheses with the mean AUC-ROC and standard error following 5-fold cross-validation with 25 repeats. The error bars indicate the standard error. lh, left hemisphere; rh, right hemisphere; pOPER, pars opercularis; LOG, lateral occipital gyrus; cACC, caudal anterior cingulate cortex; cMFG, caudal middle frontal gyrus; PeriCAL, pericalcarine; CER, cerebellum cortex; rMFG, rostral middle frontal gyrus; PTR, posterior thalamic radiation; RLIC, retrolenticular part of internal capsule; FX/ST, fornix/stria terminalis; fALFF, fractional amplitude of low-frequency fluctuations; DTI, diffusion tensor imaging; AD, axial diffusivity; NODDI, neurite orientation dispersion and density imaging; FISO, isotropic volume fraction; AUC-ROC, area under the curve for the receiver operating characteristic

To determine the most important metrics and features for SOR classification, we performed a multi-part feature selection ranking incorporating multimodal data as well as age and sex. We set our significance threshold at *p* ≤ 0.05 for all analyses. Overall, the selection ranking included fMRI (fALFF) and sMRI (volume, surface area, cortical thickness) metrics of regions (78 total, 39 per hemisphere) as well as dMRI (FA, AD, MD, RD, NDI, ODI, FISO) metrics of white matter tracts (48 total, 21 with a homologous pair and 6 without). Altogether, 49 total features were identified as being significantly important for distinguishing between ND children with and without SOR based on 5 cross-validation folds with 25 repeats. These features included 7 FCNs (Fig. 3A) and 22 regions (Fig. 3B) of fALFF, 4 tracts of DTI, 15 tracts of NODDI, and 1 region’s volume (Fig. 3C). Figure 3B and C each display their top 15 features.

By testing for optimal classification with the 7 significant FCNs (CER, SAL, VIS2, VIS1, VAN, DAN, and FPNL) from Fig. 3A, an AUC-ROC of 0.806 ± 0.106 was reached through submitting PC1 of fALFF for the CER, VIS2, VAN, and DAN to a support vector machine with a linear kernel (Fig. 3D). For a more granular analysis of fALFF, the top 15 regions shown in Fig. 3B, achieved an AUC-ROC of 0.868 ± 0.081 through PC1 of the left caudal middle frontal, right pericalcarine, left cACC, left cerebellar cortex, left caudate, left pallidum, and right rostral middle frontal regions based on a random forest classifier. Finally, the top 15 features over all metrics of gray matter regions and WM tracts (Fig. 3C) revealed that PC1 over right pars opercularis fALFF, right PTR AD, left caudal middle frontal fALFF, right pericalcarine fALFF, left cerebellar cortex fALFF, and right cerebellar cortex fALFF attained an AUC-ROC of 0.894 ± 0.074 based on a random forest classifier (Fig. 3D).

#### Long-range seed-based connectivity and structural covariance

**Fig. 4:**
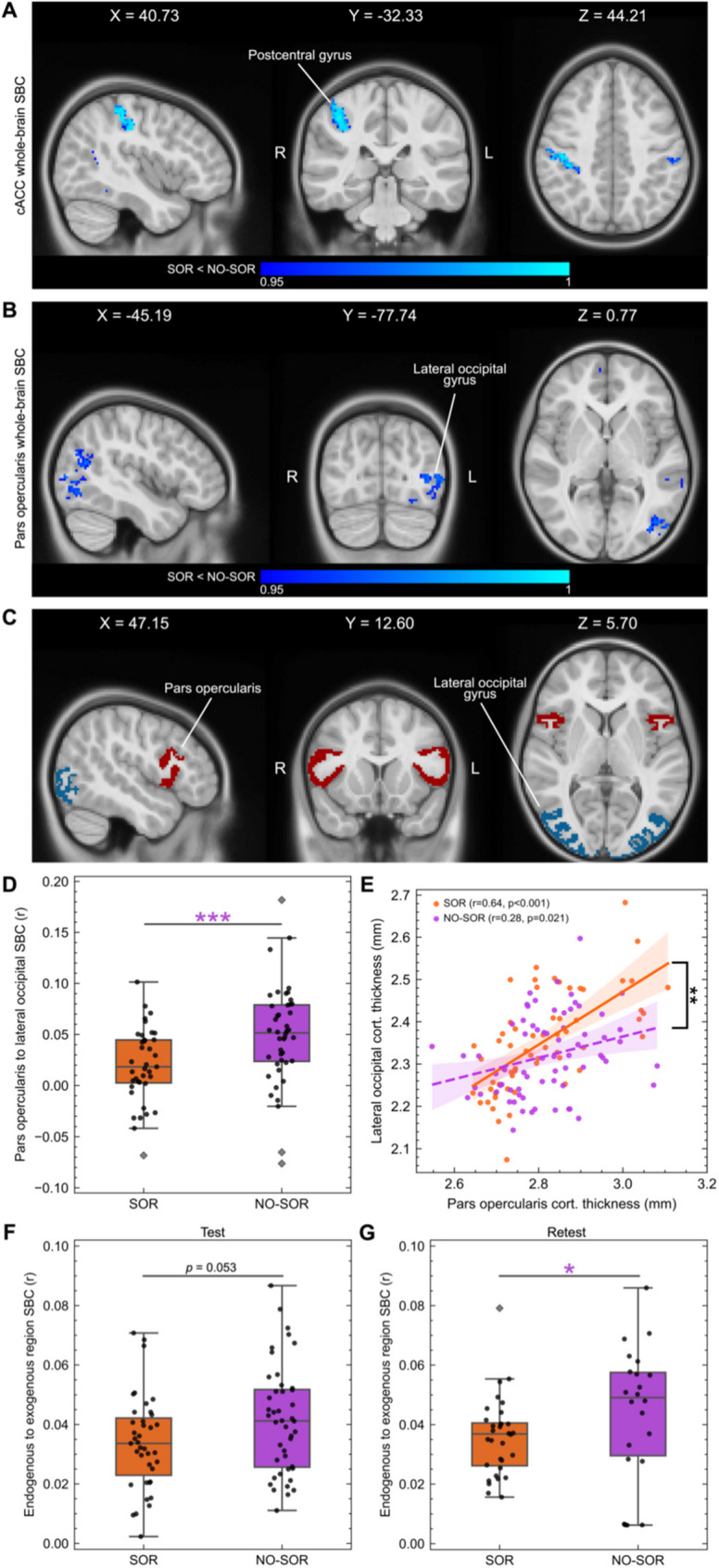
Decreased long-range seed-based connectivity and aberrant structural covariance in sensory over-responsive children. (**A**) Post hoc permutation-based voxel-wise analysis on caudal anterior cingulate cortex (cACC) and (**B**) pars opercularis whole-brain seed-based connectivity (SBC) maps. Thresholding based on *p* ≤ 0.05. World coordinates based on the *MNIPediatricAsym: cohort-4* template. (**C**) Pars opercularis in maroon (endogenous), lateral occipital gyrus in teal (exogenous). (**D**) Pars opercularis and lateral occipital SBC for test set. Boxplots delineate the median, interquartile range, and the 1.5 interquartile range, with outliers shown as gray diamonds. Post hoc Mann-Whitney U test statistical significance is indicated by asterisks: * *p* ≤ 0.05, ** *p* ≤ 0.01, and *** *p* ≤ 0.001. Asterisks are color-coded to indicate group that is significantly greater. (**E**) Pars opercularis (x-axis) and lateral occipital (y-axis) structural covariance measured by cortical thickness for 130 participants. Pearson correlation coefficient (*r*) and significance indicated in legend for ND/SOR and ND/NO-SOR groups. (**F**) *Test* and (**G**) *retest* of the mean endogenous to exogenous region-to-region SBC per participant. 20 endogenous and 14 exogenous regions formed 280 region pairs. SBC, seed-based connectivity; cACC, caudal anterior cingulate cortex; SOR and ND/SOR, neurodiverse children with sensory over-responsivity; NO-SOR and ND/NO-SOR, neurodiverse children without sensory over-responsivity

Following the identification of the bilateral pOPER, lateral occipital gyrus (LOG), cACC, and cerebellar cortex as important regions to delineate SOR status (Fig. 3, Supplementary Table 5), hypothesis-driven testing of long-range FC differences between the sensory-based cohorts was performed. This was conducted via permutation-based voxel-wise analysis of seed-based correlation (SBC) maps computed by correlating the mean time series of these bilateral regions individually with the timeseries of all other voxels within a whole-brain mask. While no significant cluster differences were found for the bilateral pOPER, LOG, and cerebellar cortex at *p* ≤ 0.025, the ND/SOR children displayed significantly weaker long-range connectivity between the cACC and postcentral gyrus, or primary somatosensory cortex (S1), in the right hemisphere at *p* ≤ 0.025 [*n voxels* = 364] but also in the left hemisphere at *p* ≤ 0.05 [*n voxels* = 915] (Fig. 4A). Interestingly, while the pOPER lacked cluster differences at *p* ≤ 0.025 [*n voxels* = 1], at *p* ≤ 0.05, the ND/SOR children showed significantly weaker long-range connectivity between the pOPER and LOG [*n voxels* = 654] (Fig. 4B). Likewise, based on notable fALFF region group differences (Supplementary Table 5) and the highest feature rankings (Fig.3, B and C), the bilateral pOPER and LOG (Fig. 4C) were investigated with region-to-region SBC analyses. In the test dataset, ND/NO-SOR youth [*Mean* = 0.05, *SD* = 0.05] showed significantly greater long-range FC [*U* = 503, *p* = 0.001, *d* = −0.67] between the bilateral pOPER and LOG than ND/SOR youth [*Mean* = 0.02, *SD* = 0.04] (Fig. 4D).

As a complement to long-range FC, structural covariance analyses of cortical thickness from 3D T1-weighted imaging were conducted for the bilateral pOPER and LOG regions, comparing between ND/SOR and ND/NO-SOR groups. Structural covariance of cortical thickness between pOPER and LOG regions was significantly stronger [*Z* = 2.500, *p* = 0.012] for ND/SOR [*r* = 0.715, *p* < 0.001] than ND/NO-SOR youth [*r* = 0.314, *p* = 0.038] based on 83 participants. Based on a larger sample of 130 participants, of which 47 additional individuals had T1-weighted imaging of acceptable quality but not fMRI, ND/SOR youth [*r* = 0.644, *p* < 0.001] exhibited significantly greater [*Z* = 2.668, *p* = 0.008] structural covariance of cortical thickness between pOPER and LOG than ND/NO-SOR youth [*r* = 0.277, *p* = 0.021] (Fig. 4E).

Regional differences showing lower long-range FC in the ND/SOR group prompted consideration of broader exogenous and endogenous brain system differences. Informed by the fALFF first principal component loading for regions, the 20 endogenous and 14 exogenous regions identified (Supplementary Fig. 2) were selected for SBC analyses to form 280 unique region pairs which were then averaged to acquire an aggregate measure of endogenous-to-exogenous long-range FC per participant. For the test set, ND/NO-SOR youth [*Mean* = 0.042, *SD* = 0.018] showed a strong trend towards greater endogenous-to-exogenous long-range FC [*U* = 645, *p* = 0.053, *d* = −0.45] than ND/SOR youth [*Mean* = 0.034, *SD* = 0.016] (Fig. 4F). Similarly, in the retest set, ND/NO-SOR youth [*Mean* = 0.044, *SD* = 0.023] showed greater endogenous-to-exogenous FC at the significance threshold [*U* = 240, *p* = 0.050, *d* = −0.46] than ND/SOR youth [*Mean* = 0.036, *SD* = 0.013] (Fig. 4G).

#### Emotional regulation behavioral clusters and associated functional connectivity by SOR status

**Fig. 5:**
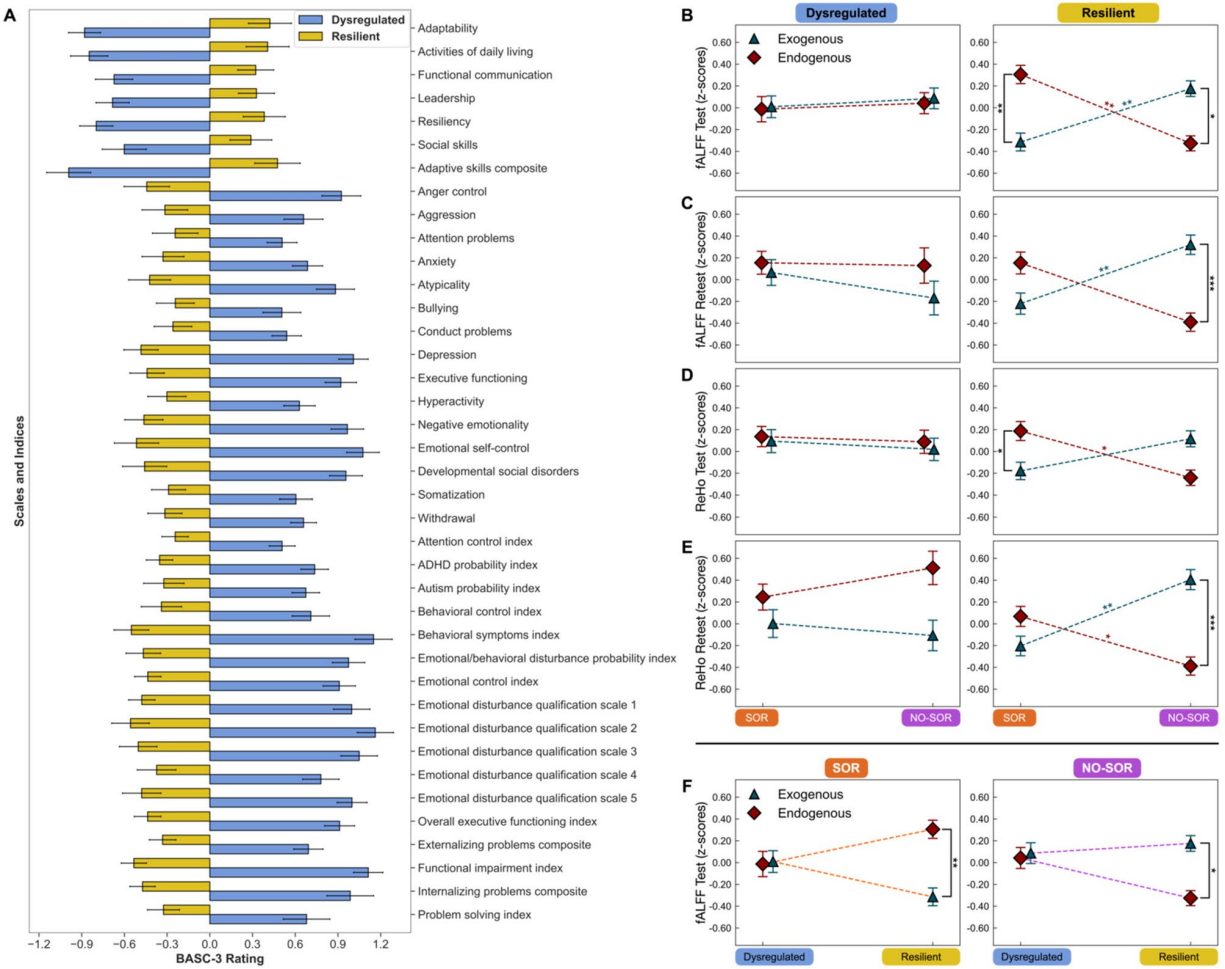
Neural signature for sensory over-responsivity and resilience defined by exogenous and endogenous brain systems. (**A**) Latent profile analysis of BASC-3 scales and indices reveals a dual behavioral construct. For the first 7 features, a higher score is associated with resilience, whereas in the following features a higher score is associated with dysregulation. For (**B** to **E**), the dysregulated cohort (left) is matched in exogenous and endogenous local functional connectivity for ND children with and without SOR. Conversely, the resilient cohort (right) exhibits a double dissociation between brain systems that is dependent on SOR status. The top 2 rows correspond to fALFF (**B**) *Test* and (**C**) *Retest*, and the bottom two rows correspond to ReHo (**D**) *Test* and (**E**) *Retest*. (**F**) Interchanged viewpoint of dysregulated and resilient exogenous and endogenous fALFF within ND/SOR and ND/NO-SOR cohorts from the test dataset. The y-axis indicates the mean FC metric value following z-scoring within participants’ whole-brain FC maps and then across participants at the network level to account for differences in the range of scores. Post hoc Mann-Whitney U test statistical significance is indicated by asterisks: * *p* ≤ 0.05, ** *p* ≤ 0.01, *** *p* ≤ 0.001. Error bars indicate standard error. BASC-3, Behavioral Assessment Scale for Children, 3rd edition; fALFF, fractional amplitude of low-frequency fluctuations; ReHo, regional homogeneity; ND, neurodiverse; SOR and ND/SOR, neurodiverse children with sensory over-responsivity; NO-SOR and ND/NO-SOR, neurodiverse children without sensory over-responsivity

Latent profile analysis (LPA) of the BASC-3 assessment (*n* = 71) identified two behavioral clusters: (1) dysregulated and (2) resilient (Fig. 5A, Supplementary Table 8). The dysregulated cluster is defined by lower scores on scales indicative of emotional reaction and behavior that is within the traditionally accepted range (e.g., adaptability and resilience) and higher scores on scales representing emotional reactions that are outside of the typical range (e.g., anger control and emotional self-control). The resilient cluster exhibits the opposite pattern. Notably, all BASC-3 measures displayed robust differences between cluster groupings following Mann-Whitney U tests with corrections for multiple comparisons (Supplementary Table 8), highlighting the effectiveness of this clustering approach in separating between resilient (positive) and dysregulated (negative) behaviors.

Of the 71 total with high quality rs-fMRI images, LPA assigned 23 children (*n* = 11 ND/SOR and *n* = 12 ND/NO-SOR) to the dysregulated cluster and 48 children (*n* = 20 ND/SOR and *n* = 28 ND/NO-SOR) to the resilient cluster. For the retest analysis, 47 children possessed a high quality second scan. In the retest analysis, 15 children (*n* = 9 ND/SOR and *n* = 6 ND/NO-SOR) were assigned to the dysregulated cluster and 32 (*n* = 17 ND/SOR and *n* = 15 ND/NO-SOR) to the resilient cluster. There was no statistical difference in framewise displacement indicative of subject motion between the dysregulated and resilient cohorts in either the test or retest fMRI analyses either as a whole or when further divided by SOR status (Supplementary Table 9).

A three-way mixed ANOVA with factors of *SOR status* (ND/SOR, ND/NO-SOR), *behavior* (dysregulated, resilient), and *network type* (exogenous, endogenous), showed trends toward an interaction between the three factors for fALFF test [*F*_(1,67)_ = 3.457, *p* = 0.067, η_*G*_^2^ = 0.036], fALFF retest [*F*_(1,43)_ = 3.672, *p* = 0.062, η_*G*_^2^ = 0.053], ReHo test [*F*_(1,67)_ = 2.047, *p* = 0.157, η_*G*_^2^ = 0.020], and ReHo retest [*F*_(1,43)_ = 5.318, *p* = 0.026, η_*G*_^2^ = 0.077] datasets (Supplementary Table 10). Of note, the resilient cluster recapitulated the double dissociation pattern seen in the overall sample with the Resilient ND/SOR cohort showing low exogenous and high endogenous FC while the Resilient ND/NO-SOR cohort displayed the inverse—high exogenous and low endogenous FC (Fig. 5B to F; Supplementary Table 11). This pattern was consistent across both local FC approaches, fALFF and ReHo, in test and retest analyses. Critically, FC for resilience is diametrically opposed based on whether the child is sensory over-responsive.

For dysregulated ND children, both the SOR and NO-SOR subgroups displayed matched levels of exogenous and endogenous local FC (Fig. 5B to F; Supplementary Table 11). Figure 5F and Supplementary Figure 7 alongside Supplementary Table 12 offer an important complementary view of the local FC results, showcasing the behavioral clusters and their associated exogenous and endogenous local FC within the ND/SOR and ND/NO-SOR groups.

## Discussion

**Fig. 6:**
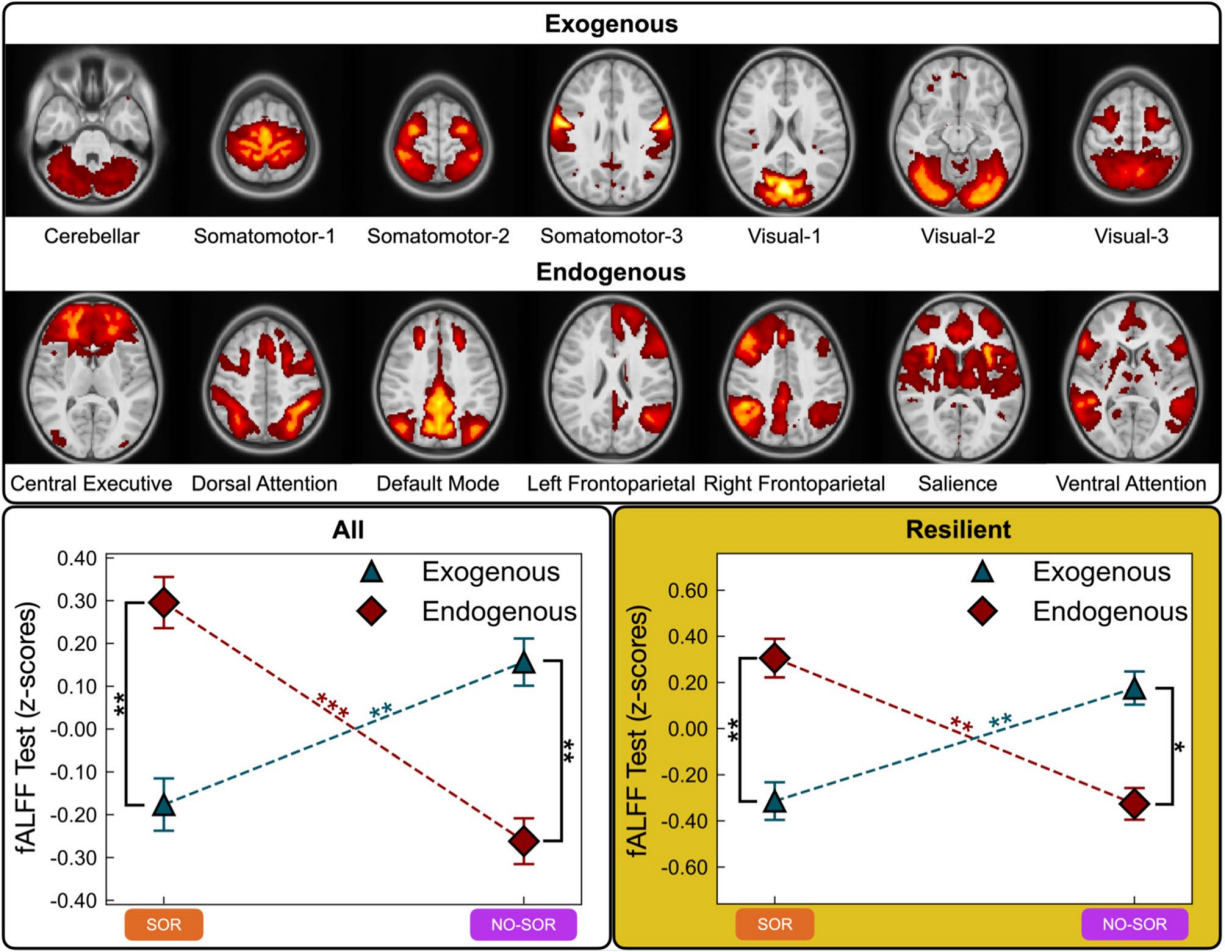
Main findings as a graphical abstract. (Top) The dichotomy between exogenous and endogenous networks exhibits (Bottom Left) a double dissociation in FC between our ND/SOR and ND/NO-SOR populations where ND/SOR children display reduced exogenous FC but elevated endogenous FC. (Bottom Right) This double dissociation is unique to resilient youth and suggests contrasting regulatory mechanisms between resilient ND/SOR and ND/NO-SOR youth. fALFF, fractional amplitude of low-frequency fluctuations; SOR and ND/SOR, neurodiverse children with sensory over-responsivity; NO-SOR and ND/NO-SOR, neurodiverse children without sensory over-responsivity

Employing a data-driven approach, we found that dual exogenous and endogenous brain systems best represent functional connectivity for neurodiverse children with and without SOR. Additionally, this work has created a testable scientific premise for characterizing emotional regulation using functional brain activity. Furthermore, we found that a matched contrast whereby the exogenous and endogenous brain systems’ FC are equally and oppositely split signifies behavioral resilience and that the direction of this complementary contrast is specific to SOR (Fig. 6). Thus, the SOR phenotype is critical to understanding the neural mechanisms of resilience in neurodiverse children.

On a macro level, this work utilizes sensory over-responsivity, the most clinically relevant and robust feature of the sensory framework [80], to move from a structural to a functional understanding of neural mechanisms. We add a new dimension to the previous structural finding of decreased white matter integrity [17, 18, 81, 82] by showing long-range and local exogenous functional hypoconnectivity in networks, lobes, and regions. Second, this work successfully utilizes the high intensity phenotype of interest, SOR, atop a noisy neural background to achieve new understanding that is directly applicable to the clinical population it was derived from—all comers to a neurodevelopmental clinic at high risk for a neurodevelopmental disorder. By sequentially recruiting 8- to 12-year-old children, we create a matched background of neurodiversity phenotypes and by extension, neurotypes, upon which to probe which differences are specific to SOR. This allows the findings herein to be isolated to the impact of SOR and generalized more readily to this clinical population. Third, this work builds upon the extensive neuropsychology and neuroimaging work attempting to create an overarching framework of neural systems by asking if brain tracts or networks can be best understood by “lumping” into simplified constructs or “systems”, recognizing that there is a role both for splitting and lumping in clinical care and research.

### Exogenous functional connectivity is reduced in sensory over-responsive children

On a micro level, our research reveals a consistent reduction in FC, or hypoconnectivity, across the exogenous networks for sensory over-responsive children, encompassing both long-range (DR) and local connectivity (fALFF, ReHo) metrics. This observation of reduced exogenous FC is congruent with prior dMRI research studies of SPD children, generally [17, 81, 82], and SOR specifically [18]. The existing literature identified deficient WM microstructure within cerebral sensory tracts and cortico-cerebellar pathways which is echoed by the present study’s noted reduction in the visual networks (VIS1, VIS2), manifested across all assessed metrics (DR, fALFF, and ReHo), and the visuo-cerebellar network (CER) which is most notable for local FC (fALFF). It also agrees with rs-fMRI results from the ABCD cohort in which SBC analyses of sensorimotor network nodes showed reduced FC in children at risk for SOR [16]. The ABCD study, however, was not designed to investigate sensory processing disorders; thus, the sensory cohort was determined by a single parent-report item from the Social Responsiveness Scale [83]: “Seems overly sensitive to sounds, textures, or smells”. As the authors state, this might explain why the sensorimotor network alone, as opposed to more widespread impact, was emphasized in the ABCD results. In our sensory-focused neuroimaging investigation, we employed a comprehensive, clinician-led, direct assessment for SOR via the SP3D:A and whole-brain rs-fMRI network decomposition that reveal a broader array of exogenous FC changes despite the smaller sample size. More importantly, however, this study highlights the complementary contrast, a double dissociation, between exogenous and endogenous brain system FC that is dependent on SOR phenotype.

### Sensory-based double dissociation of local brain system connectivity in neurodiverse children

A double dissociation emerges for local FC (fALFF and ReHo) between exogenous and endogenous FCNs, highlighting the critical difference in neural connectivity for ND children with and without SOR. Specifically, the data reveal that ND children with SOR not only show lower FC in the exogenous brain system but striking elevation in the endogenous networks. By contrast, the ND children without SOR, display an inverse FC presentation—high exogenous and low endogenous. Dual systems for brain organization and function, including the “top-down/bottom-up” [84, 85], “excitation/inhibition” [86], and “local overconnectivity and long-range underconnectivity” models [87], are important theoretical drivers that have transformed neuroscience and neurodevelopmental research and clinical care over the past century. However, which regions, tracts, or networks belong in which aspects of the duality remains an open question.

An example of the permeability of common terms is the “top-down” dorsal attention network (DAN) versus the “bottom-up” ventral attention network (VAN) model [88–90] whereby these two functional networks are contrasting entities. However, in our data-driven approach, both DAN and VAN are lumped into the endogenous brain system. This corresponds to a recent rs-fMRI investigation of VAN in childhood brain development showing that advancing FC among VAN regions correlated with maturation of higher-order cognition, as would be expected of top-down rather than bottom-up networks [91]. Our local FC results at a network level, highlighting the importance of the VAN, also agree with the SBC analysis from the ABCD cohort identifying greater VAN FC in children at risk for SOR [16]. Our data-driven analysis of local FC extends this knowledge by showing a stronger and more spatially extensive alteration of endogenous networks in SOR than the more limited ABCD study hypothesis-driven SBC approach and provides a unifying dual brain system FC model to be further explored in the future.

Moving from networks to regions, the lateral occipital cortices are the foci of greatest voxel-wise differences in data-driven whole-brain comparisons over all FC metrics in the ND/SOR cohort relative to the ND/NO-SOR cohort. There is also a suggestion of hemispheric connectivity differences in conjunction with the results of region-wise group differences and feature importance of SOR, in which the right lateral occipital cortex is the most important exogenous system feature. It is particularly noteworthy that the right lateral occipital cortex has been linked to psychological measures of resilience in a prior sMRI study of adults [92] and a separate sMRI study of children [93]. Both studies suggest that emotional processing of visual inputs along the ventral processing stream is the underlying mechanism. Our finding of reduced long-range FC between bilateral occipital cortex and pars opercularis of the inferior frontal lobe for the ND/SOR children across multiple SBC approaches also supports the notion of dysfunction in the ventral visual processing stream [94]. Interestingly, structural covariance between the two regions across participants was higher for the ND children with SOR than those without. Although structural covariance is often interpreted as greater connectivity when observed within participants, the wider range of low and high cortical thicknesses across ND/SOR children suggests that abnormal cortical morphology in lateral occipital cortex is more likely to be mirrored in pars opercularis for ND/SOR children than ND/NO-SOR children. The intricate relationship suggested between the pars opercularis and lateral occipital cortex from our post hoc examination of regional connectivity specific to SOR status calls for further investigation.

In addition to region and network FC, SBC analysis of long-range FC shows weaker communication between exogenous and endogenous regions in general for ND/SOR children, as observed between the pars opercularis and lateral occipital cortex. A notable decrease in cACC (endogenous) to postcentral (exogenous) FC revealed for ND/SOR children suggests weak connectivity between regions involved in somatosensory prediction/perception and those involved in processing/response. Existing literature indicates that enhancement in long-range, large-scale connectivity alongside a diminution of short-range connectivity is expected as a part of healthy middle childhood brain development in both FC and white matter structural connectivity [95–98]. This developmental pattern of strengthening FCNs over time has also been reported for control networks, frontoparietal and cinguloopercular, including connections with the cerebellum [99]. The contrary pattern of low long-range FC and structural connectivity identified in our study of SOR youth may represent an additional marker for atypical neurodevelopment over time which requires a longitudinal approach to further decipher.

Attentional challenges are a common clinical complaint and co-morbid condition in neurodiverse children in general and previously reported in 40% of children with SPD [15]. An additional nuance, however, is that hyperactivity in the SAL network, particularly within the anterior insula, may intensify the assignment of salience to internal and external events, leading to excessive detection of incoming sensory information that might be counterproductive for an individual’s best cognitive and behavioral functioning. The SAL network has been referred to as a toggle between the CEN and DMN, broadly mediating between systems of activation (“cognitively demanding mental activity”) and rest (“self-referential mental activity”). However, in the case of SAL dysfunction, a problematic bias toward a state of activation may encourage further hyperresponsiveness [20, 100, 101]. Consequently, a down-regulation of exogenous networks, as seen in the ND/SOR cohort, might be required to mitigate an onslaught of exogenous sensory input. On the other hand, for ND children, particularly those with internally driven anxiety, who do not experience SOR, an inverse relationship between endogenous and exogenous networks would be expected to be beneficial. Overall, our findings suggest that a subject-specific balance is needed between exogenous and endogenous systems, supported by the Triple Network Model which asserts that a core network dysfunction can have cascading effects on other networks, potentially manifesting in clinical symptoms [102–104]. Additional ecological assessment of the autonomic nervous system would clarify our understanding of these FC observations and inform interventions.

In our analysis, the best-fit model for the FCNs is a two-factor model, and the FCNs align well in general with the sensorimotor–association axis framework such that the exogenous networks are closer to the sensorimotor pole while the endogenous networks are members of the association pole. It may be interesting, or surprising, to note that the cerebellar network, a recognized motor, speech, and attention modulation hub [105], falls within the exogenous brain system while the default mode network, which has limbic contributions, sits squarely within the endogenous system when letting the data inform the research learning. However, the cerebellar network in this data-driven FCN specification relies heavily on visual cortex connections and has a foundational role in the prediction of incoming sensorimotor information which may explain its alignment. The segmenting of the FCNs into dual brain systems (e.g., exogenous and exogenous) is not a novel concept, however, this work operationalizes the construct in a neurodiverse pediatric cohort using a data-driven delineation of the resting state fMRI networks based on local FC variation for both the test and retest imaging samples. It highlights a critical difference for ND children with SOR, and from this overarching dual brain system construct, we are then able to construe hemispheric and regional differences which inform clinical relevance.

### Moving from brain systems to resilience

Especially striking is that the complementary FC brain system contrast—equal and opposite elevation and reduction—is evidenced only in ND children who are resilient based on parent report of behavior in the real world. This double dissociation is nonexistent at the group level for emotionally dysregulated children. This was found for both fALFF and ReHo in the test analysis and validated in the retest cohort. The low local FC of exogenous FCNs and their component regions for the ND/SOR children is concordant with observed low long-range FC and disrupted WM connectivity. However, resilient individuals in the ND/SOR group have even lower exogenous local FC than those who are dysregulated, again suggesting that reduced local connectivity in sensory-related cerebral cortex and cerebellum may be an adaptive response to sensory over-responsivity. Conversely, the higher local FC of the endogenous system for the ND/SOR resilient children indicates that elevated connectivity within higher-order attention, salience, and cognitive control networks is also adaptive.

### Extrapolating SOR long-range and local connectivity findings to autistic individuals

In this study, long-range FC underconnectivity in SOR is corroborated by reduced long-range seed-based correlation between nodes of the endogenous and exogenous systems in fMRI and reduced microstructural integrity of long-range WM tracts in dMRI. The reduced long-range and local exogenous connectivity in SOR but elevated local connectivity of endogenous networks may be related to the long-standing concept of short-range overconnectivity and long-range underconnectivity as a hallmark of ASD [106, 107]. A recent rs-fMRI study of child, adolescent, and adult males with ASD has confirmed long-range underconnectivity but has also shown short-range overconnectivity particularly in paralimbic and heteromodal cortex of the prefrontal and anterior temporal lobes [108]. These regions of short-range overconnectivity correspond to nodes of the SAL, VAN, and CEN networks, which are all endogenous by our classification. However, the short-range fMRI measure used in that study differs from our local FC metrics such that direct comparison is not possible.

Children meeting research criteria for autism were excluded from our study because the sample size was too small for conclusive statistical inferences and may also increase the heterogeneity of the sample. Furthermore, children with SPD but not ASD are very understudied, especially with advanced neuroimaging, despite outnumbering children with clinical ASD [109, 110]. Therefore, we feel that our fMRI study of SOR in a non-ASD cohort addresses an important gap in the current literature. More work with harmonized or complementary approaches is needed to learn whether the present findings of endogenous system local overconnectivity and more diffuse long-range underconnectivity in SOR generalize to clinical ASD cohorts.

### SOR classification and future directions

A potential clinical application is highlighted by exploratory machine learning classification with cross-validation that distinguished children with SOR with 87% accuracy based on their resting state fALFF FCN signature. When including white matter microstructural information, classification accuracy approaches 90%. Notably, the two most important white matter tracts for SOR classification are the posterior thalamic radiations, which contain the optic radiations, and the retrolenticular internal capsule, which contains the somatosensory radiations, specifically the right-sided tracts for both. The two top cortical regions for classification are the right pars opercularis—part of the two best network-level endogenous features (SAL and VAN) as well as DAN, FPNL (both significant network-level endogenous features), and FPNR—and the right lateral occipital cortex, which is a central part of the second-best network-level exogenous feature (VIS2). It is possible that these right-sided structures are favored over their left hemispheric homologues given the right hemispheric specialization for visuospatial processing in the human brain bearing in mind that the children were resting but had their eyes open [111]. This is an area for further investigation. Although further research in larger and more diverse cohorts is needed to establish whether these high classification accuracies generalize to other populations, the ramifications are significant given that SOR appears likely to impact treatment approach for emotional resilience at a neural level and resting state neuroimaging is feasible in clinical practice for many affected individuals. Over time, understanding the relationship between neuroimaging metrics, electroencephalographic signatures, and wearable physiological metrics will bring this knowledge out of the lab and into clinical care.

It remains to be determined from longitudinal studies spanning infancy to adulthood when and how adaptive (resilient) versus maladaptive (dysregulated) brain connectivity profiles emerge and evolve over time. Large population-based studies with dense genotyping, neuroimaging, and sensory, cognitive, and behavioral phenotyping will elucidate monogenic, polygenic, and epigenetic components as well as factors responsive to environmental changes, targeted developmental therapies, neuromodulation, and neurochemistry. To study if the local FC findings of resilience represent compensatory neuroplasticity, interventional trials to remediate SOR could be performed with pre- and post-treatment neuroimaging. We close in mentioning that we are currently working to examine the interaction of SPD and ADHD, given our previous dMRI work [60] and the notable overlap between these conditions in our sample; collect the data to reproduce these findings in an independent cohort; and generalize our findings to mesoscale resolution fMRI using a state-of-the-art 7 Tesla MRI scanner with next-generation gradient and radiofrequency coil performance as well as novel pulse sequences for faster scanning.

## Conclusions

Herein, we describe a unique neural substrate for sensory over-responsivity using resting state fMRI. We propose a dual brain system construct that separates the major functional connectivity networks of the human brain into an exogenous sensory-oriented system and an endogenous higher-order system such that neurodiverse children with SOR show a distinct pattern of low exogenous activity and high endogenous connectivity and those without SOR show the opposite, a double dissociation. This dichotomy among rs-fMRI networks is consistent with recent work on the macroscale organization of the human cerebral cortex along a sensorimotor–association axis but also generalizes the SA axis distinction to subcortical structures such as the basal ganglia and cerebellum. Moreover, the proper balance of local functional activity between exogenous and endogenous systems appears crucial for developing emotional resilience, and the direction of this complementary contrast is specific to SOR. Given that the exogenous system is the first to develop, and that the later-developing endogenous system requires robust and well-tuned sensory input from the exogenous system, this study’s findings hold substantial implications for understanding the neural mechanisms of neurodiversity in the pursuit of providing the right support for individuals to achieve resilience.

## Supplementary Information

Supplementary Material

## Data Availability

All data needed to evaluate the conclusions in the paper are present in the paper and/or the Supplementary Materials. The datasets presented in this study can be found in online repositories at the NIH Data Archive (https://nda.nih.gov) through Accession Number: 4095004.

## Abbreviations

3T: 3 Tesla
ABCD: Adolescent brain cognitive development
AD: Axial diffusivity
ADHD: Attention deficit hyperactivity disorder
ALFF: Amplitude of low-frequency fluctuations
AP: Anterior to posterior
ASD: Autism spectrum disorder
AUC-ROC: Area under the curve for the receiver operating characteristic
BASC-3: Behavioral assessment scale for children, 3rd edition
BH: Benjamini-Hochberg
C-PAC: Configurable pipeline for the analysis of connectomes
cACC: Caudal anterior cingulate cortex
CEN: Central executive network
CER: Cerebellar
CSF: Cerebrospinal fluid
DAN: Dorsal attention network
DKT: Desikan-Killiany-Tourville
DMN: Default mode network
dMRI: Diffusion magnetic resonance imaging
DR: Dual regression
DTI: Diffusion tensor imaging
ESSENCE-Q-REV: Early symptomatic syndromes eliciting neurodevelopmental clinical examinations-questionnaire-revised
FA: Fractional anisotropy
fALFF: Fractional amplitude of low-frequency fluctuations
FAST: FMRIB’s automated segmentation tool
FC: Functional connectivity
FCN: Functional connectivity network
FD: Framewise displacement
FDR: False discovery rate
FISO: Isotropic volume fraction
fMRI: Functional magnetic resonance imaging
FMRIB: Functional magnetic resonance imaging of the brain
FPNL: Left frontoparietal network
FPNR: Right frontoparietal network
FSL: FMRIB software library
FWHM: Full width at half maximum
FX/ST: Fornix/stria terminalis
GLM: General linear model
GM: Gray matter
GNB: Gaussian naïve bayes
ICA-AROMA: Independent component analysis-automatic removal of motion artifacts
KCC: Kendall’s coefficient of concordance
KNN: K-nearest neighbors
LDA: Linear discriminant analysis
LOG: Lateral occipital gyrus
LPA: Latent profile analysis
MD: Mean diffusivity
MELODIC: Multivariate exploratory linear optimized decomposition into independent components
ND: Neurodiverse
ND/NO-SOR: Neurodiverse without sensory over-responsivity
ND/SOR: Neurodiverse with sensory over-responsivity
NDI: Neurite density index
NODDI: Neurite orientation dispersion and density imaging
ODI: Orientation dispersion index
PA: Posterior to anterior
PC1: First principal component
PCA: Principal component analysis
pOPER: Pars opercularis
PTR: Posterior thalamic radiation
RBF: Radial basis function
RD: Radial diffusivity
ReHo: Regional homogeneity
RLIC: Retrolenticular part of internal capsule
ROI: Region of interest
rs-fMRI: Resting state fMRI
S1: Somatosensory cortex
SA: Sensorimotor to association
SAL: Salience network
SBC: Seed-based connectivity/correlation
SBCA: Seed-based connectivity/correlation analyses
SMN1: Somatomotor-1 network
SMN2: Somatomotor-2 network
SMN3: Somatomotor-3 network
sMRI: Structural magnetic resonance imaging
SOR: Sensory over-responsivity
SP3D:A: Sensory processing 3 dimensions assessment
SPD: Sensory processing disorder
SUSAN: Smallest univalue segment assimilating nucleus
SVM: Support vector machine
T1w: T1-weighted
TFCE: Threshold-free cluster enhancement
UCSF: University of California, San Francisco
VAN: Ventral attention network
VIS1: Visual-1 network
VIS2: Visual-2 network
VIS3: Visual-3 network
WISC-V: Wechsler intelligence scale for children, 5th edition
WM: White matter
