# Supplementary material for: A neural substrate for sensory over-responsivity defined by exogenous and endogenous brain systems
Source: J Neurodev Disord. 2025 Nov 21;17:68. doi: 10.1186/s11689-025-09656-y (PMC12636187; doi:10.1186/s11689-025-09656-y)
Supplement: Supplementary file 1 — Supplementary Material [file 11689_2025_9656_MOESM1_ESM.docx]

Supplementary Materials for

**A neural substrate for sensory over-responsivity defined by exogenous and endogenous brain systems**

Hannah L. Choi, Maia C. Lazerwitz, Rachel Powers, Mikaela Rowe, Jamie Wren-Jarvis, Amir Sadikov, Lanya T. Cai, Robyn Chu, LaShelle Rullan, Kaitlyn J. Trimarchi, Rafael D. Garcia, Elysa J. Marco* and Pratik Mukherjee*

*Corresponding authors. Elysa J. Marco, [drmarco@linccenter.com](mailto:drmarco@linccenter.com); Pratik Mukherjee, [pratik.mukherjee@ucsf.edu](mailto:pratik.mukherjee@ucsf.edu)

**This PDF file includes:**

Supplementary Text

Supplementary Figures 1 to 7

Supplementary Tables 1 to 12

Supplementary Text

fMRIPrep: anatomical data, field map, and functional data preprocessing

The text provided below, detailing the processes involved in anatomical data, field map, and functional data preprocessing, was automatically generated by *fMRIPrep* (*37*) for the explicit purpose of inclusion in user manuscripts. The material is made available under the CC0 license.

Anatomical data preprocessing

The T1w image was corrected for intensity non-uniformity (INU) with N4BiasFieldCorrection (*112*), distributed with ANTs 2.3.3 (*113*), and used as a T1w-reference throughout the workflow. The T1w-reference was then skull-stripped with a *Nipype* implementation of the antsBrainExtraction.sh workflow (from ANTs), using NKI as the target template. Brain tissue segmentation of cerebrospinal fluid (CSF), white-matter (WM), and gray-matter (GM) was performed on the brain-extracted T1w using fast (FSL 6.0.5.1:57b01774) (*54*). Brain surfaces were reconstructed using recon-all (*57*) (FreeSurfer 7.3.2), and the brain mask estimated previously was refined with a custom variation of the method to reconcile ANTs-derived and FreeSurfer-derived segmentations of the cortical gray-matter of Mindboggle (*114*). Volume-based spatial normalization to MNIPediatricAsym:cohort-4 standard space was performed through nonlinear registration with antsRegistration (ANTs 2.3.3), using brain-extracted versions of both the T1w reference and the T1w template. The following template was selected for spatial normalization and accessed with *TemplateFlow* (*55*) (23.0.0): *MNI’s unbiased standard MRI template for pediatric data from the 7.5 to 13.5y age range*, TemplateFlow ID: MNIPediatricAsym:cohort-4.

Preprocessing of B_0_ inhomogeneity mappings

A *B_0_*-nonuniformity map (or *fieldmap*) was estimated based on two echo-planar imaging (EPI) references with topup (FSL 6.0.5.1:57b01774) (*115*).

Functional data preprocessing

For each of the BOLD runs found per subject (across all tasks and sessions), the following preprocessing was performed. First, a reference volume and its skull-stripped version were generated using a custom methodology of *fMRIPrep*. Head-motion parameters with respect to the BOLD reference (transformation matrices, and six corresponding rotation and translation parameters) are estimated before any spatiotemporal filtering using mcflirt (FSL 6.0.5.1:57b01774) (*116*). The estimated *fieldmap* was then aligned with rigid-registration to the target EPI (echo-planar imaging) reference run. The field coefficients were mapped onto the reference EPI using the transform.

The BOLD reference was then co-registered to the T1w reference using bbregister (FreeSurfer) which implements boundary-based registration (*117*). Co-registration was configured with six degrees of freedom. Several confounding time-series were calculated based on the *preprocessed BOLD*: framewise displacement (FD), DVARS, and three region-wise global signals. FD was computed using two formulations: absolute sum of relative motions (*118*) and relative root mean square displacement between affines (*116*). FD and DVARS are calculated for each functional run, both using their implementations in *Nipype* (*118*). The three global signals are extracted within the CSF, the WM, and the whole-brain masks.

Additionally, a set of physiological regressors were extracted to allow for component-based noise correction (*CompCor)* (*119*)*.* Principal components are estimated after high-pass filtering the *preprocessed BOLD* time-series (using a discrete cosine filter with 128s cut-off) for the two *CompCor* variants: temporal (tCompCor) and anatomical (aCompCor). tCompCor components are then calculated from the top 2% variable voxels within the brain mask. For aCompCor, three probabilistic masks (CSF, WM, and combined CSF+WM) are generated in anatomical space. The implementation differs from that of *CompCor* in that instead of eroding the masks by 2 pixels on BOLD space, a mask of pixels that likely contain a volume fraction of GM is subtracted from the aCompCor masks. This mask is obtained by dilating a GM mask extracted from the FreeSurfer’s *aseg* segmentation, and it ensures components are not extracted from voxels containing a minimal fraction of GM. Finally, these masks are resampled into BOLD space and binarized by thresholding at 0.99 (as in the original implementation). Components are also calculated separately within the WM and CSF masks. For each CompCor decomposition, the *k* components with the largest singular values are retained, such that the retained components’ time series are sufficient to explain 50 percent of variance across the nuisance mask (CSF, WM, combined, or temporal). The remaining components are dropped from consideration. The head-motion estimates calculated in the correction step were also placed within the corresponding confounds file. The confound time series derived from head motion estimates and global signals were expanded with the inclusion of temporal derivatives and quadratic terms for each (*120*). Frames that exceeded a threshold of 0.5mm FD or 1.5 standardized DVARS were annotated as motion outliers. Additional nuisance timeseries are calculated by means of principal component analysis of the signal found within a thin band (*crown*) of voxels around the edge of the brain (*121*).

The BOLD time-series were resampled into standard space, generating a *preprocessed BOLD run in MNIPediatricAsym:cohort-4 space*. First, a reference volume and its skull-stripped version were generated using a custom methodology of *fMRIPrep*. Automatic removal of motion artifacts using independent component analysis (ICA-AROMA) (*42*) was performed on the *preprocessed BOLD on MNI space* time-series after removal of non-steady state volumes and spatial smoothing with an isotropic, Gaussian kernel of 6mm FWHM (full-width half-maximum). Corresponding “non-aggressively” denoised runs were produced after such smoothing. Additionally, the “aggressive” noise-regressors were collected and placed in the corresponding confounds file.

All resamplings can be performed with *a single interpolation step* by composing all the pertinent transformations (i.e., head-motion transform matrices, susceptibility distortion correction when available, and co-registrations to anatomical and output spaces). Gridded (volumetric) resamplings were performed using antsApplyTransforms (ANTs), configured with Lanczos interpolation to minimize the smoothing effects of other kernels (*122*). Non-gridded (surface) resamplings were performed using mri_vol2surf (FreeSurfer).

Supplementary Figure 1. Local whole-brain gray matter functional connectivity comparing neurodiverse children with and without SOR. (A) Whole-brain gray matter (GM) mask created using FMRIB’s Automated Segmentation Tool (FAST) segmentation of the *MNIPediatricAsym:cohort-4* standard space template, tailored for subjects aged 7.5–13.5 years. (B) Test and (C) Retest sets. Local FC metrics are on the x-axis. The y-axis represents the mean value of the voxels within whole-brain GM for the FC measure where voxel-wise z-scoring was applied within participants’ whole-brain FC maps. Boxplots delineate the median, interquartile range, and the 1.5 interquartile range, with outliers shown as gray diamonds. GM, gray matter; fALFF, fractional amplitude of low-frequency fluctuations; ReHo, regional homogeneity; SOR, neurodiverse children with sensory over-responsivity; NO-SOR, neurodiverse children without sensory over-responsivity.

**Supplementary Figure 2. fALFF first principal component loading across lobes and bilateral regions.** First principal component loading across lobes, bilateral regions, and participants for (**A**) test and (**B**) retest sets. Lobes (visualized in Supplementary Fig. 3) are labeled on top and separated by dotted lines. Regions corresponding to the lobes, as defined in the Appendix of Klein & Tourville (2012) (*62*), are indicated on the bottom with matching color schemes. The y-axis represents the loading of the first principal component (PC1). Exogenous regions are assigned teal, and endogenous regions are assigned maroon if directionality is consistent between test and retest datasets. Regions with ambiguous directionality, inconsistent between datasets, are assigned gray. The explained variance ratio of PC1 for the test set is 24.7% and 22.7% for the retest set. fALFF, fractional amplitude of low-frequency fluctuations; Lat. Temp., Lateral Temporal; Med. Temp., Medial Temporal; AHCx, amygdalohippocampal complex; In., Insula; Cer., Cerebellum.

Supplementary Figure 3. Lobe gray matter masks. Lobe masks are delineated according to the Appendix of Klein and Tourville (2012) (*59*) and displayed on the *MNIPediatricAsym:cohort-4* standard template.

**Supplementary Figure 4. Retest: data driven determination of exogenous and endogenous brain systems from canonical functional connectivity networks.** (**A**) Resting state functional MRI data for all participants generated 14 stable functional connectivity networks based on MELODIC probability estimates for each voxel’s association with specific independent components. These networks have been subjected to thresholding, Gaussian smoothing with a full width half maximum (FWHM) kernel of 4mm for the purpose of visualization, and are displayed on top of the *MNIPediatricAsym:cohort-4* standard template. They are grouped by exogenous (left) and endogenous (right) brain systems. (**B**) First principal component loading, denoted on the y-axis, across long-range and local functional connectivity metrics explains 26.2% of the variance. FC, functional connectivity; MELODIC, multivariate exploratory linear optimized decomposition into independent components; fALFF, fractional amplitude of low-frequency fluctuations; ReHo, regional homogeneity.

**Supplementary Figure 5. Functional connectivity network correlation matrices across metrics.** Correlation matrices are displayed for functional connectivity metrics: Dual Regression, fALFF, and ReHo within both (**A**) test and (**B**) retest sets. FCNs represent both axes. They have been grouped into exogenous (top and left) and endogenous (bottom and right) networks. A color bar indicating the range of correlation coefficients is provided on the top right with warmer colors indicating positive correlations and cooler colors denoting negative or anti-correlations. Asterisks mark statistically significant correlations, corrected for multiple comparisons: **p* ≤ 0.05, ***p* ≤ 0.01, and ****p* ≤ 0.001. fALFF, fractional amplitude of low-frequency fluctuations; ReHo, regional homogeneity; FCN, functional connectivity network.

**Supplementary Figure 6. Test: ND/SOR versus ND/NO-SOR across functional connectivity metrics and FCNs.** (**A**) Dual Regression, (**B**) fALFF, and (**C**) ReHo are displayed top to bottom. FCNs are on the x-axis grouped into exogenous (left) and endogenous (right) networks. The y-axis represents the mean value of the voxels within an FCN, where z-scoring was first applied within participants’ whole-brain FC maps voxel-wise and then across subjects at the network level to account for differences in the range of scores. Boxplots delineate the median, interquartile range, and the 1.5 interquartile range, with outliers shown as diamonds. Each point represents a participant. Statistical significance, adjusted for multiple comparisons, is indicated by asterisks: * *p* ≤ 0.05, ** *p* ≤ 0.01, and *** *p* ≤ 0.001. Asterisks are color-coded to indicate the group that is significantly greater. fALFF, fractional amplitude of low-frequency fluctuations; ReHo, regional homogeneity; SOR and ND/SOR, neurodiverse children with sensory over-responsivity; NO-SOR and ND/NO-SOR, neurodiverse children without sensory over-responsivity; FCN, functional connectivity network.

**Supplementary Figure 7. ND/SOR and ND/NO-SOR cohorts defined by emotional regulation and exogenous and endogenous local functional connectivity.** (**A** to **D**) The resilient ND/SOR cohort (left) exhibits greater endogenous over exogenous local FC. Conversely, the resilient ND/NO-SOR cohort (right) exhibits greater exogenous over endogenous local FC. The dysregulated cohort (blue) is matched in exogenous and endogenous local FC for ND children with and without SOR. The top 2 rows correspond to fALFF (**A)** *Test* and (**B**) *Retest*, and the bottom two rows correspond to ReHo (**C**) *Test* and (**D**) *Retest*. The y-axis indicates the mean FC metric value following z-scoring within participants’ whole-brain FC maps and then across participants at the network level to account for differences in the range of scores. Post hoc Mann-Whitney U test statistical significance is indicated by asterisks: * *p* ≤ 0.05, ** *p* ≤ 0.01, and *** *p* ≤ 0.001. Error bars indicate standard error. FC, functional connectivity; fALFF, fractional amplitude of low-frequency fluctuations; ReHo, regional homogeneity; SOR and ND/SOR, neurodiverse children with sensory over-responsivity; NO-SOR and ND/NO-SOR, neurodiverse children without sensory over-responsivity; ND, neurodiverse.

Supplementary Table 1.

**2x2 ANOVAs with factors *SOR* *status* (SOR, NO-SOR) and *Network* *type* (EXO, ENDO).**

| **FC Metric** | **Set** | ***Effect*** | ***DFn*** | ***DFd*** | ***SSn*** | ***SSd*** | ***F*** | ***p*** | $\boldsymbol{\eta}$***_G_^2^*** |
| --- | --- | --- | --- | --- | --- | --- | --- | --- | --- |
| **Dual Regression** | **Test**  **(n = 83)** | ***(Intercept)*** | 1 | 81 | 0.016 | 51.663 | 0.024 | 0.876 | 0.000 |
|  |  | ***SOR vs. NO-SOR*** | 1 | 81 | 4.272 | 51.663 | 6.699 | **0.011*** | **0.061** |
|  |  | ***EXO vs. ENDO*** | 1 | 81 | 0.000 | 14.473 | 0.000 | 0.993 | 0.000 |
|  |  | ***SOR/NO-SOR x EXO/ENDO*** | 1 | 81 | 0.003 | 14.473 | 0.019 | 0.891 | 0.000 |
|  | **Retest**  **(n = 54)** | ***(Intercept)*** | 1 | 52 | 0.070 | 41.738 | 0.087 | 0.769 | 0.001 |
|  |  | ***SOR vs. NO-SOR*** | 1 | 52 | 2.038 | 41.738 | 2.539 | 0.117 | 0.039 |
|  |  | ***EXO vs. ENDO*** | 1 | 52 | 0.010 | 7.987 | 0.063 | 0.803 | 0.000 |
|  |  | ***SOR/NO-SOR x EXO/ENDO*** | 1 | 52 | 0.281 | 7.987 | 1.829 | 0.182 | 0.006 |
| **fALFF** | **Test**  **(n = 83)** | ***(Intercept)*** | 1 | 81 | 0.002 | 21.544 | 0.007 | 0.933 | 0.000 |
|  |  | ***SOR vs. NO-SOR*** | 1 | 81 | 0.522 | 21.544 | 1.962 | 0.165 | 0.007 |
|  |  | ***EXO vs. ENDO*** | 1 | 81 | 0.030 | 51.063 | 0.047 | 0.829 | 0.000 |
|  |  | ***SOR/NO-SOR x EXO/ENDO*** | 1 | 81 | 8.191 | 51.063 | 12.993 | **0.001***** | **0.101** |
|  | **Retest**  **(n = 54)** | ***(Intercept)*** | 1 | 52 | 0.006 | 15.338 | 0.022 | 0.883 | 0.000 |
|  |  | ***SOR vs. NO-SOR*** | 1 | 52 | 0.187 | 15.338 | 0.634 | 0.430 | 0.004 |
|  |  | ***EXO vs. ENDO*** | 1 | 52 | 0.117 | 29.097 | 0.208 | 0.650 | 0.003 |
|  |  | ***SOR/NO-SOR x EXO/ENDO*** | 1 | 52 | 3.402 | 29.097 | 6.080 | **0.017*** | **0.071** |
| **ReHo** | **Test**  **(n = 83)** | ***(Intercept)*** | 1 | 81 | 0.001 | 21.520 | 0.004 | 0.953 | 0.000 |
|  |  | ***SOR vs. NO-SOR*** | 1 | 81 | 0.258 | 21.520 | 0.970 | 0.328 | 0.004 |
|  |  | ***EXO vs. ENDO*** | 1 | 81 | 0.015 | 40.674 | 0.030 | 0.862 | 0.000 |
|  |  | ***SOR/NO-SOR x EXO/ENDO*** | 1 | 81 | 4.218 | 40.674 | 8.400 | **0.005**** | **0.064** |
|  | **Retest**  **(n = 54)** | ***(Intercept)*** | 1 | 52 | 0.003 | 13.387 | 0.011 | 0.919 | 0.000 |
|  |  | ***SOR vs. NO-SOR*** | 1 | 52 | 0.079 | 13.387 | 0.308 | 0.582 | 0.002 |
|  |  | ***EXO vs. ENDO*** | 1 | 52 | 0.098 | 26.270 | 0.194 | 0.661 | 0.002 |
|  |  | ***SOR/NO-SOR x EXO/ENDO*** | 1 | 52 | 2.858 | 26.270 | 5.657 | **0.021*** | **0.067** |

*Note.* Summary of two-way mixed ANOVAs based on a 2x2 factorial design conducted for each FC metric: dual regression, fALFF, and ReHo within both test and retest sets. The between-subjects factor was *SOR status* (SOR, NO-SOR), and the within-subjects factor was *network type* (exogenous, endogenous). We display the effects being examined, degrees of freedom for the numerator (*DFn*) and denominator (*DFd*), sum of squares for the numerator (*SSn*) and denominator (*SSd*), F-value, p-value, and generalized eta squared ($\eta$*_G_^2^*). All values are rounded to three decimal places. FC, functional connectivity; fALFF, fractional amplitude of low-frequency fluctuations; ReHo, regional homogeneity; SOR, neurodiverse children with sensory over-responsivity; NO-SOR, neurodiverse children without sensory over-responsivity; EXO, exogenous; ENDO, endogenous.

Supplementary Table 2.

**Comparisons for *SOR status* (SOR, NO-SOR) x *Network type* (Exo, Endo) across all FC metrics.**

| **Set** | **FC Metric** | **Grouped By** | **Comparison** | **Mann-Whitney *U*** | **p-value** | **Cohen’s *d*** |
| --- | --- | --- | --- | --- | --- | --- |
| **Test**  **(n = 83)** | **Dual Regression** | SOR | Exo vs. Endo | 724 | 0.719 | -0.02 |
|  |  | NO-SOR |  | 965 | 0.983 | 0.01 |
|  |  | Exogenous | SOR vs. NO-SOR | 599 | **0.018*** | **-0.52** |
|  |  | Endogenous |  | 605 | **0.021*** | **-0.49** |
|  | **fALFF** | SOR | Exo vs. Endo | 456 | **0.002**** | **-0.69** |
|  |  | NO-SOR |  | 1324 | **0.003**** | **0.64** |
|  |  | Exogenous | SOR vs. NO-SOR | 567 | **0.008**** | **-0.59** |
|  |  | Endogenous |  | 1222 | **0.001***** | **0.73** |
|  | **ReHo** | SOR | Exo vs. Endo | 485 | **0.006**** | **-0.57** |
|  |  | NO-SOR |  | 1231 | **0.028*** | **0.47** |
|  |  | Exogenous | SOR vs. NO-SOR | 637 | **0.044*** | **-0.43** |
|  |  | Endogenous |  | 1168 | **0.005**** | **0.59** |
| **Retest**  **(n = 54)** | **Dual Regression** | SOR | Exo vs. Endo | 438 | 0.324 | -0.12 |
|  |  | NO-SOR |  | 267 | 0.565 | 0.18 |
|  |  | Exogenous | SOR vs. NO-SOR | 236 | **0.042*** | **-0.55** |
|  |  | Endogenous |  | 306 | 0.423 | -0.26 |
|  | **fALFF** | SOR | Exo vs. Endo | 370 | 0.057 | -0.44 |
|  |  | NO-SOR |  | 347 | **0.014*** | **0.68** |
|  |  | Exogenous | SOR vs. NO-SOR | 238 | **0.046*** | **-0.51** |
|  |  | Endogenous |  | 471 | **0.037*** | **0.59** |
|  | **ReHo** | SOR | Exo vs. Endo | 346 | **0.026*** | **-0.46** |
|  |  | NO-SOR |  | 337 | **0.027*** | **0.60** |
|  |  | Exogenous | SOR vs. NO-SOR | 205 | **0.010**** | **-0.73** |
|  |  | Endogenous |  | 465 | **0.048*** | **0.40** |

*Note.* The p-value following post hoc Mann-Whitney U tests and effect size indicated by Cohen’s *d* are reported. A positive Cohen’s *d* would indicate that EXO > ENDO for Exo vs. Endo comparisons or SOR > NO-SOR for SOR vs. NO-SOR comparisons, while a negative Cohen’s *d* would indicate that ENDO > EXO or NO-SOR > SOR. Statistical significance is indicated by asterisks: * *p* ≤ 0.05, ** *p* ≤ 0.01, and *** *p* ≤ 0.001. FC, functional connectivity; fALFF, fractional amplitude of low-frequency fluctuations; ReHo, regional homogeneity; SOR, neurodiverse children with sensory over-responsivity; NO-SOR, neurodiverse children without sensory over-responsivity; EXO, exogenous; ENDO, endogenous.

Supplementary Table 3.

**Test: SOR versus NO-SOR groups across FC measures and FCNs.**

| **FC Metric** | **FCN** | **SOR: Mean (SD)** | **NO-SOR: Mean (SD)** | **Mann-Whitney *U*** | **p-value** | **Adjusted p-value** | **Cohen’s *d*** |
| --- | --- | --- | --- | --- | --- | --- | --- |
| **Dual Regression** | CER | -0.08 (0.91) | 0.07 (1.09) | 763 | 0.389 | 0.566 | -0.14 |
|  | SMN1 | -0.09 (1.08) | 0.08 (0.94) | 777 | 0.463 | 0.566 | -0.17 |
|  | SMN2 | -0.18 (0.99) | 0.16 (1.00) | 687 | 0.120 | 0.280 | -0.35 |
|  | SMN3 | -0.10 (1.01) | 0.09 (1.01) | 762 | 0.384 | 0.566 | -0.18 |
|  | VIS1 | -0.33 (1.00) | 0.29 (0.93) | 538 | **0.004**** | **0.022*** | **-0.64** |
|  | VIS2 | -0.32 (0.89) | 0.28 (1.03) | 547 | **0.005**** | **0.022*** | **-0.62** |
|  | VIS3 | -0.13 (0.86) | 0.11 (1.12) | 774 | 0.446 | 0.566 | -0.24 |
|  | CEN | -0.39 (0.92) | 0.34 (0.97) | 498 | **0.001***** | **0.015*** | **-0.77** |
|  | DAN | -0.04 (0.85) | 0.03 (1.13) | 796 | 0.575 | 0.575 | -0.07 |
|  | DMN | -0.09 (1.00) | 0.08 (1.02) | 781 | 0.485 | 0.566 | -0.17 |
|  | FPNL | -0.20 (0.75) | 0.18 (1.17) | 657 | 0.067 | 0.189 | -0.38 |
|  | FPNR | -0.06 (0.88) | 0.05 (1.11) | 788 | 0.526 | 0.566 | -0.11 |
|  | SAL | -0.28 (1.02) | 0.25 (0.93) | 584 | **0.013*** | **0.044*** | **-0.55** |
|  | VAN | -0.11 (0.94) | 0.09 (1.07) | 778 | 0.468 | 0.566 | -0.20 |
| **fALFF** | CER | -0.48 (0.89) | 0.42 (0.92) | 420 | **<0.001***** | **0.001***** | **-1.00** |
|  | SMN1 | 0.00 (1.07) | -0.00 (0.95) | 839 | 0.866 | 0.866 | 0.01 |
|  | SMN2 | 0.03 (1.06) | -0.03 (0.96) | 892 | 0.760 | 0.818 | 0.06 |
|  | SMN3 | 0.08 (1.06) | -0.07 (0.97) | 944 | 0.435 | 0.553 | 0.15 |
|  | VIS1 | -0.38 (0.97) | 0.33 (0.92) | 508 | **0.001***** | **0.004**** | **-0.75** |
|  | VIS2 | -0.43 (0.94) | 0.38 (0.91) | 440 | **<0.001***** | **0.001***** | **-0.88** |
|  | VIS3 | -0.07 (0.96) | 0.06 (1.05) | 779 | 0.474 | 0.553 | -0.13 |
|  | CEN | 0.27 (1.02) | -0.24 (0.94) | 1113 | **0.020*** | **0.035*** | **0.52** |
|  | DAN | 0.32 (0.92) | -0.28 (1.00) | 1138 | **0.011*** | **0.022*** | **0.63** |
|  | DMN | 0.17 (0.97) | -0.15 (1.03) | 1054 | 0.074 | 0.104 | 0.33 |
|  | FPNL | 0.31 (1.03) | -0.27 (0.91) | 1169 | **0.005**** | **0.011*** | **0.60** |
|  | FPNR | 0.21 (1.03) | -0.18 (0.96) | 1068 | 0.056 | 0.087 | 0.40 |
|  | SAL | 0.43 (1.07) | -0.38 (0.78) | 1287 | **<0.001***** | **0.001***** | **0.88** |
|  | VAN | 0.35 (0.94) | -0.31 (0.97) | 1246 | **<0.001***** | **0.001***** | **0.70** |
| **ReHo** | CER | -0.19 (1.09) | 0.17 (0.91) | 685 | 0.116 | 0.202 | -0.36 |
|  | SMN1 | -0.09 (1.01) | 0.08 (1.01) | 794 | 0.562 | 0.656 | -0.17 |
|  | SMN2 | 0.01 (0.97) | -0.01 (1.05) | 881 | 0.837 | 0.837 | 0.03 |
|  | SMN3 | -0.09 (0.86) | 0.08 (1.12) | 776 | 0.457 | 0.625 | -0.17 |
|  | VIS1 | -0.32 (1.08) | 0.28 (0.85) | 552 | **0.005**** | **0.025*** | **-0.62** |
|  | VIS2 | -0.36 (0.95) | 0.32 (0.95) | 529 | **0.003**** | **0.019*** | **-0.70** |
|  | VIS3 | 0.14 (0.79) | -0.12 (1.16) | 934 | 0.491 | 0.625 | 0.26 |
|  | CEN | -0.06 (0.95) | 0.05 (1.06) | 830 | 0.802 | 0.837 | -0.11 |
|  | DAN | 0.30 (0.84) | -0.27 (1.07) | 1148 | **0.008**** | **0.029*** | **0.59** |
|  | DMN | 0.22 (0.95) | -0.19 (1.02) | 1045 | 0.089 | 0.178 | 0.42 |
|  | FPNL | 0.39 (0.97) | -0.35 (0.92) | 1236 | **0.001***** | **0.008**** | **0.78** |
|  | FPNR | 0.26 (1.02) | -0.23 (0.95) | 1129 | **0.014*** | **0.038*** | **0.49** |
|  | SAL | 0.15 (1.06) | -0.13 (0.95) | 1010 | 0.167 | 0.260 | 0.28 |
|  | VAN | 0.22 (1.01) | -0.19 (0.97) | 1093 | **0.032*** | 0.076 | 0.42 |

*Note.* This table reflects the test set where n = 83. For each functional connectivity (FC) measure, functional connectivity networks (FCNs) have been grouped into exogenous networks (top) and endogenous networks (bottom). Summary statistics—including the mean and standard deviation for each group, p-value following Mann-Whitney U tests, adjusted p-value following Benjamini-Hochberg corrections, and effect size indicated by Cohen’s *d*—are reported. Statistical significance is indicated by asterisks: * *p* ≤ 0.05, ** *p* ≤ 0.01, and *** *p* ≤ 0.001. FC, functional connectivity; FCN, functional connectivity network; fALFF, fractional amplitude of low-frequency fluctuations; ReHo, regional homogeneity; SOR, neurodiverse children with sensory over-responsivity; NO-SOR, neurodiverse children without sensory over-responsivity.

Supplementary Table 4.

**fALFF: SOR versus NO-SOR groups across lobes.**

| **Set** | **Lobe** | **SOR: Mean (SD)** | **NO-SOR: Mean (SD)** | **Mann-Whitney *U*** | **p-value** | **Adjusted p-value** | **Cohen’s *d*** |
| --- | --- | --- | --- | --- | --- | --- | --- |
| **Test**  **(n = 83)** | **Frontal** | 0.40 (1.01) | -0.35 (0.87) | 1241 | **<0.001***** | **0.002**** | **0.80** |
|  | **Occipital** | -0.41 (0.99) | 0.37 (0.88) | 481 | **0.001***** | **0.002**** | **-0.84** |
|  | **Cerebellum** | -0.38 (0.94) | 0.34 (0.95) | 513 | **0.002**** | **0.004**** | **-0.76** |
|  | **Medial Temporal** | -0.19 (1.05) | 0.17 (0.94) | 672 | 0.091 | 0.181 | -0.36 |
|  | **Cingulate** | 0.18 (1.09) | -0.16 (0.91) | 1029 | 0.120 | 0.192 | 0.34 |
|  | **Insula** | 0.19 (1.04) | -0.17 (0.96) | 994 | 0.216 | 0.288 | 0.35 |
|  | **Lateral Temporal** | 0.10 (1.05) | -0.09 (0.96) | 959 | 0.359 | 0.410 | 0.20 |
|  | **Parietal** | -0.08 (0.97) | 0.07 (1.04) | 798 | 0.587 | 0.587 | -0.14 |
| **Retest**  **(n = 54)** | **Frontal** | 0.16 (1.09) | -0.23 (0.85) | 424 | 0.208 | 0.333 | 0.38 |
|  | **Occipital** | -0.20 (1.08) | 0.28 (0.84) | 256 | 0.093 | 0.333 | -0.48 |
|  | **Cerebellum** | -0.17 (1.03) | 0.24 (0.95) | 257 | 0.096 | 0.333 | -0.41 |
|  | **Medial Temporal** | -0.10 (1.10) | 0.15 (0.86) | 298 | 0.346 | 0.462 | -0.25 |
|  | **Cingulate** | 0.15 (0.91) | -0.22 (1.13) | 429 | 0.178 | 0.333 | 0.38 |
|  | **Insula** | 0.12 (0.93) | -0.18 (1.11) | 397 | 0.433 | 0.495 | 0.30 |
|  | **Lateral Temporal** | 0.09 (1.16) | -0.13 (0.75) | 357 | 0.937 | 0.937 | 0.21 |
|  | **Parietal** | -0.08 (1.02) | 0.11 (1.01) | 279 | 0.202 | 0.333 | -0.18 |

*Note.* For the test set, lobes have been ordered based on the adjusted p-value. In cases where the adjusted p-values were identical, the ordering was further refined by the uncorrected p-value in ascending order. The ordering of lobes for the retest set reflects the test set to facilitate comparison. Summary statistics—including the mean and standard deviation for each group, p-value following Mann-Whitney U tests, adjusted p-value following Benjamini-Hochberg corrections, and effect size indicated by Cohen’s *d*—are reported. Statistical significance is indicated by asterisks: * *p* ≤ 0.05, ** *p* ≤ 0.01, and *** *p* ≤ 0.001. fALFF, fractional amplitude of low-frequency fluctuations; SOR, neurodiverse children with sensory over-responsivity; NO-SOR, neurodiverse children without sensory over-responsivity.

Supplementary Table 5.

**fALFF: SOR versus NO-SOR groups across regions.**

| **Region** | **SOR:**  **Mean (SD)** | **NO-SOR: Mean (SD)** | **Mann-Whitney *U*** | **p-value** | **Adjusted p-value** | **Cohen’s *d*** |
| --- | --- | --- | --- | --- | --- | --- |
| **rh.ParsOpercularis** | 0.49 (0.95) | -0.43 (0.85) | 1309 | **<0.001***** | **0.003**** | **1.03** |
| **rh.LateralOccipital** | -0.47 (0.96) | 0.41 (0.87) | 430 | **<0.001***** | **0.004**** | **-0.96** |
| **rh.CaudalAnteriorCingulate** | 0.43 (0.99) | -0.38 (0.87) | 1264 | **<0.001***** | **0.005**** | **0.87** |
| **lh.CaudalMiddleFrontal** | 0.42 (0.96) | -0.37 (0.90) | 1255 | **<0.001***** | **0.005**** | **0.84** |
| **lh.Amygdala** | 0.29 (0.69) | -0.26 (1.17) | 1250 | **<0.001***** | **0.005**** | **0.57** |
| **lh.Caudate** | 0.37 (0.93) | -0.33 (0.96) | 1247 | **<0.001***** | **0.005**** | **0.73** |
| **lh.LateralOccipital** | -0.41 (0.98) | 0.37 (0.89) | 471 | **<0.001***** | **0.005**** | **-0.83** |
| **rh.PeriCalcarine** | -0.40 (1.01) | 0.35 (0.87) | 498 | **0.001***** | **0.010**** | **-0.80** |
| **lh.ParsOpercularis** | 0.35 (0.98) | -0.31 (0.93) | 1207 | **0.001***** | **0.013*** | **0.70** |
| **lh.CaudalAnteriorCingulate** | 0.38 (1.06) | -0.34 (0.83) | 1204 | **0.002**** | **0.013*** | **0.77** |
| **lh.CerebellumCortex** | -0.38 (0.90) | 0.33 (0.98) | 519 | **0.002**** | **0.013*** | **-0.75** |
| **rh.CerebellumCortex** | -0.36 (0.97) | 0.32 (0.94) | 519 | **0.002**** | **0.013*** | **-0.72** |
| **lh.PeriCalcarine** | -0.38 (1.02) | 0.33 (0.88) | 522 | **0.002**** | **0.013*** | **-0.75** |
| **lh.SuperiorFrontal** | 0.32 (1.01) | -0.28 (0.93) | 1166 | **0.005**** | **0.025*** | **0.63** |
| **rh.ParsTriangularis** | 0.32 (1.07) | -0.28 (0.86) | 1165 | **0.005**** | **0.025*** | **0.62** |
| **rh.SuperiorFrontal** | 0.32 (0.99) | -0.28 (0.95) | 1164 | **0.005**** | **0.025*** | **0.62** |
| **rh.Lingual** | -0.34 (1.02) | 0.30 (0.90) | 556 | **0.006**** | **0.025*** | **-0.67** |
| **lh.Pallidum** | 0.37 (0.75) | -0.33 (1.09) | 1160 | **0.006**** | **0.025*** | **0.73** |
| **rh.RostralMiddleFrontal** | 0.33 (1.01) | -0.30 (0.92) | 1159 | **0.006**** | **0.025*** | **0.66** |
| **rh.Fusiform** | -0.30 (1.06) | 0.27 (0.89) | 565 | **0.008**** | **0.030*** | **-0.58** |
| **rh.Cuneus** | -0.29 (1.03) | 0.25 (0.92) | 576 | **0.010**** | **0.038*** | **-0.55** |
| **rh.Amygdala** | 0.26 (0.75) | -0.23 (1.15) | 1137 | **0.011*** | **0.038*** | **0.49** |
| **lh.Entorhinal** | 0.27 (1.00) | -0.24 (0.96) | 1136 | **0.011*** | **0.038*** | **0.53** |
| **rh.Caudate** | 0.24 (1.02) | -0.21 (0.95) | 1133 | **0.012*** | **0.040*** | **0.46** |
| **lh.Lingual** | -0.30 (1.00) | 0.26 (0.94) | 593 | **0.016*** | **0.049*** | **-0.58** |

*Note.* For brevity, only regions with significant group differences after Benjamini-Hochberg corrections are displayed. For a full list of regions, please see Supplementary Fig. 5. Regions have been ordered based on the adjusted p-value. In cases where the adjusted p-values were identical, the ordering was further refined by the uncorrected p-value in ascending order. Summary statistics—including the mean and standard deviation for each group, p-value following Mann-Whitney U tests, adjusted p-value following Benjamini-Hochberg corrections, and effect size indicated by Cohen’s *d*—are reported. Statistical significance is indicated by asterisks: * *p* ≤ 0.05, ** *p* ≤ 0.01, and *** *p* ≤ 0.001. rh, right hemisphere; lh, left hemisphere; fALFF, fractional amplitude of low-frequency fluctuations; SOR, neurodiverse children with sensory over-responsivity; NO-SOR, neurodiverse children without sensory over-responsivity.

Supplementary Table 6.

**Whole-brain gray matter local functional connectivity between SOR and NO-SOR groups.**

| **Set** | **FC Metric** | **SOR:**  **Mean (SD)** | **NO-SOR:**  **Mean (SD)** | **Mann-Whitney *U*** | **p-value** | **Cohen’s *d*** |
| --- | --- | --- | --- | --- | --- | --- |
| **Test**  **(n = 83)** | **fALFF** | 0.23 (0.02) | 0.23 (0.03) | 821 | 0.739 | -0.01 |
|  | **ReHo 7** | 0.05 (0.03) | 0.05 (0.04) | 928 | 0.526 | 0.07 |
|  | **ReHo 19** | 0.09 (0.03) | 0.09 (0.04) | 881 | 0.837 | -0.01 |
|  | **ReHo 27** | 0.11 (0.03) | 0.11 (0.04) | 862 | 0.975 | -0.04 |
| **Retest**  **(n = 54)** | **fALFF** | 0.23 (0.03) | 0.23 (0.03) | 353 | 0.993 | 0.05 |
|  | **ReHo 7** | 0.04 (0.04) | 0.05 (0.03) | 314 | 0.509 | -0.19 |
|  | **ReHo 19** | 0.08 (0.04) | 0.09 (0.03) | 304 | 0.403 | -0.27 |
|  | **ReHo 27** | 0.10 (0.04) | 0.11 (0.03) | 295 | 0.320 | -0.30 |

*Note.* Summary statistics—including the mean and standard deviation for each group, p-value following Mann-Whitney U tests, and effect size indicated by Cohen’s *d*—are reported for test and retest sets. FC, functional connectivity; fALFF, fractional amplitude of low-frequency fluctuations; ReHo, regional homogeneity; SOR, neurodiverse children with sensory over-responsivity; NO-SOR, neurodiverse children without sensory over-responsivity. 7, 19, and 27 indicate cluster size based on number of voxels.

Supplementary Table 7.

**DTI and NODDI analysis for white matter tracts between SOR and NO-SOR groups.**

| **Metric** | **Tract** | **SOR:**  **Mean (SD)** | **NO-SOR:**  **Mean (SD)** | **Mann-Whitney *U*** | **p-value** | **Adjusted p-value** | **Cohen’s *d*** |
| --- | --- | --- | --- | --- | --- | --- | --- |
| **DTI**  **(AD)** | **rh.PTR** | 1.448E-03 (4.4E-05) | 1.488E-03 (4.4E-05) | 440 | **<0.001***** | **0.007**** | **-0.93** |
|  | **rh.RLIC** | 1.258E-03 (4.6E-05) | 1.292E-03 (3.4E-05) | 483 | **0.001***** | **0.015*** | **-0.85** |
| **NODDI**  **(FISO)** | **lh.FX/ST** | 0.13 (0.02) | 0.15 (0.02) | 485 | **0.001***** | **0.032*** | **-0.79** |
|  | **lh.PTR** | 0.12 (0.02) | 0.13 (0.02) | 529 | **0.003**** | **0.049*** | **-0.71** |
|  | **rh.RLIC** | 0.13 (0.02) | 0.14 (0.02) | 533 | **0.003**** | **0.049*** | **-0.62** |

*Note.* For brevity, only tracts with significant group differences following Benjamini-Hochberg corrections are displayed. Lateralized tracts based on the Johns Hopkins University (JHU) White Matter (WM) Tractography Atlas have been ordered according to the adjusted p-value. In cases where the adjusted p-values were identical, the ordering was further refined by the unadjusted p-value in ascending order. Summary statistics—including the mean and standard deviation for each group, p-value following Mann-Whitney U tests, adjusted p-value following Benjamini-Hochberg corrections, and effect size indicated by Cohen’s *d*—are reported. Statistical significance is indicated by asterisks: * *p* ≤ 0.05, ** *p* ≤ 0.01, and *** *p* ≤ 0.001. DTI, Diffusion Tensor Imaging; AD, axial diffusivity (in mm^2^/sec); NODDI, Neurite Orientation Dispersion and Density Imaging; FISO, isotropic volume fraction (dimensionless); rh, right hemisphere; lh, left hemisphere; PTR, posterior thalamic radiation; RLIC, retrolenticular part of internal capsule; FX/ST, fornix/stria terminalis; SOR, neurodiverse children with sensory over-responsivity; NO-SOR, neurodiverse children without sensory over-responsivity.

Supplementary Table 8.

**Breakdown of dysregulated and resilient behavioral clusters by BASC-3 features.**

| **Feature (Scale/Index)** | **Higher Score** | **Dysregulated: Mean (SD)** | **Resilient: Mean (SD)** | **Mann-Whitney *U*** | **p-value** | **Adjusted p-value** | **Cohen’s *d*** |
| --- | --- | --- | --- | --- | --- | --- | --- |
| Adaptability | **+** | -0.89 (0.59) | 0.40 (0.87) | 126.5 | **< 0.001***** | **< 0.001***** | **-1.64** |
| Activities of daily living |  | -0.86 (0.64) | 0.38 (0.87) | 144 | **< 0.001***** | **< 0.001***** | **-1.54** |
| Functional communication |  | -0.69 (0.68) | 0.31 (0.97) | 215 | **< 0.001***** | **< 0.001***** | **-1.13** |
| Leadership |  | -0.70 (0.61) | 0.31 (0.98) | 216.5 | **< 0.001***** | **< 0.001***** | **-1.14** |
| Resiliency |  | -0.81 (0.53) | 0.36 (0.94) | 139 | **< 0.001***** | **< 0.001***** | **-1.41** |
| Social skills |  | -0.62 (0.95) | 0.27 (0.89) | 280 | **0.001***** | **0.001***** | **-0.97** |
| Adaptive skills composite |  | -1.00 (0.61) | 0.45 (0.76) | 75 | **< 0.001***** | **< 0.001***** | **-2.02** |
| Anger control | **-** | 0.94 (0.92) | -0.44 (0.70) | 978.5 | **< 0.001***** | **< 0.001***** | **1.77** |
| Aggression |  | 0.67 (1.13) | -0.30 (0.76) | 841.5 | **< 0.001***** | **< 0.001***** | **1.09** |
| Attention problems |  | 0.52 (0.90) | -0.23 (0.97) | 781 | **0.005**** | **0.005**** | **0.80** |
| Anxiety |  | 0.69 (0.92) | -0.33 (0.87) | 875.5 | **< 0.001***** | **< 0.001***** | **1.15** |
| Atypicality |  | 0.89 (1.05) | -0.42 (0.65) | 958 | **< 0.001***** | **< 0.001***** | **1.64** |
| Bullying |  | 0.52 (1.16) | -0.23 (0.82) | 764.5 | **0.008**** | **0.008**** | **0.80** |
| Conduct problems |  | 0.54 (1.13) | -0.27 (0.83) | 791.5 | **0.003**** | **0.003**** | **0.86** |
| Depression |  | 1.02 (1.00) | -0.48 (0.54) | 1038 | **< 0.001***** | **< 0.001***** | **2.09** |
| Executive functioning |  | 0.93 (0.69) | -0.41 (0.80) | 1007.5 | **< 0.001***** | **< 0.001***** | **1.76** |
| Hyperactivity |  | 0.63 (0.89) | -0.30 (0.92) | 850.5 | **< 0.001***** | **< 0.001***** | **1.03** |
| Negative emotionality |  | 0.98 (1.00) | -0.45 (0.59) | 1006.5 | **< 0.001***** | **< 0.001***** | **1.92** |
| Emotional self-control |  | 1.09 (0.89) | -0.50 (0.53) | 1073.5 | **< 0.001***** | **< 0.001***** | **2.40** |
| Developmental social disorders |  | 0.97 (0.88) | -0.45 (0.69) | 982 | **< 0.001***** | **< 0.001***** | **1.88** |
| Somatization |  | 0.61 (1.10) | -0.29 (0.82) | 814 | **0.001***** | **0.001***** | **0.99** |
| Withdrawal |  | 0.67 (1.06) | -0.30 (0.81) | 839 | **< 0.001***** | **< 0.001***** | **1.08** |
| Attention control index |  | 0.52 (0.90) | -0.23 (0.97) | 781 | **0.005**** | **0.005**** | **0.80** |
| ADHD probability index |  | 0.75 (0.76) | -0.34 (0.91) | 906 | **< 0.001***** | **< 0.001***** | **1.26** |
| Autism probability index |  | 0.69 (1.21) | -0.31 (0.69) | 830 | **0.001***** | **0.001***** | **1.12** |
| Behavioral control index |  | 0.71 (0.87) | -0.34 (0.88) | 891.5 | **< 0.001***** | **< 0.001***** | **1.19** |
| Behavioral symptoms index |  | 1.16 (0.64) | -0.54 (0.59) | 1086.5 | **< 0.001***** | **< 0.001***** | **2.82** |
| EBD probability index |  | 0.99 (0.91) | -0.46 (0.64) | 1005 | **< 0.001***** | **< 0.001***** | **1.96** |
| Emotional control index |  | 0.91 (1.00) | -0.44 (0.65) | 946 | **< 0.001***** | **< 0.001***** | **1.73** |
| Emotional disturbance qualification scale composite 1 |  | 1.01 (0.80) | -0.46 (0.68) | 1015 | **< 0.001***** | **< 0.001***** | **2.03** |
| Emotional disturbance qualification scale composite 2 |  | 1.18 (0.64) | -0.55 (0.57) | 1091 | **< 0.001***** | **< 0.001***** | **2.91** |
| Emotional disturbance qualification scale composite 3 |  | 1.06 (0.90) | -0.50 (0.57) | 1052 | **< 0.001***** | **< 0.001***** | **2.26** |
| Emotional disturbance qualification scale composite 4 |  | 0.79 (0.87) | -0.38 (0.84) | 928 | **< 0.001***** | **< 0.001***** | **1.38** |
| Emotional disturbance qualification scale composite 5 |  | 1.01 (0.75) | -0.46 (0.71) | 1036.5 | **< 0.001***** | **< 0.001***** | **2.04** |
| Overall executive functioning index |  | 0.91 (0.78) | -0.44 (0.78) | 994.5 | **< 0.001***** | **< 0.001***** | **1.73** |
| Externalizing problems composite |  | 0.70 (1.04) | -0.33 (0.81) | 868 | **< 0.001***** | **< 0.001***** | **1.16** |
| Functional impairment index |  | 1.13 (0.58) | -0.52 (0.66) | 1075.5 | **< 0.001***** | **< 0.001***** | **2.60** |
| Internalizing problems composite |  | 1.00 (0.85) | -0.48 (0.67) | 1015.5 | **< 0.001***** | **< 0.001***** | **2.01** |
| Problem solving index |  | 0.70 (0.50) | -0.30 (1.01) | 916 | **< 0.001***** | **< 0.001***** | **1.14** |

*Note.* 71 of 83 children with a functional scan adequate for analysis had a complete BASC-3 assessment. Following latent profile analysis, 23 children were assigned to the dysregulated cluster and 48 to the resilient cluster. 31 children were SOR and 40 were NO-SOR. Dysregulated: SOR (n = 11, 47.83%), NO-SOR (n = 12, 52.17%); Resilient: SOR (n = 20, 41.67%), NO-SOR (n = 28, 58.33%). In the higher score column, a + indicates that a higher score for the feature is associated with resilient (positive) behaviors, while a – indicates that a higher score for the feature is associated with dysregulated (negative) behaviors. Summary statistics—including the mean and standard deviation for each cluster, p-value following Mann-Whitney U tests, adjusted p-value following Benjamini-Hochberg corrections, and effect size indicated by Cohen’s *d*—are reported. Statistical significance is indicated by asterisks: * *p* ≤ 0.05, ** *p* ≤ 0.01, and *** *p* ≤ 0.001. BASC-3, Behavior Assessment System for Children, 3rd edition; SOR, neurodiverse children with sensory over-responsivity; NO-SOR, neurodiverse children without sensory over-responsivity.

Supplementary Table 9.

**Framewise displacement breakdown between dysregulated and resilient clusters over participant groupings.**

| **Set** | **SOR Status** | **Dysregulated:**  **Mean (SD)** | **Resilient:**  **Mean (SD)** | **Mann-Whitney *U*** | **p-value** | **Cohen’s *d*** |
| --- | --- | --- | --- | --- | --- | --- |
| **Test**  **(n = 71)** | **All** | 0.21 (0.10) | 0.22 (0.08) | 459.5 | 0.258 | -0.17 |
|  | **SOR** | 0.19 (0.06) | 0.19 (0.06) | 103 | 0.788 | -0.07 |
|  | **NO-SOR** | 0.22 (0.12) | 0.24 (0.09) | 135 | 0.337 | -0.18 |
| **Retest**  **(n = 47)** | **All** | 0.23 (0.10) | 0.25 (0.09) | 216 | 0.592 | -0.13 |
|  | **SOR** | 0.23 (0.08) | 0.23 (0.10) | 74 | 0.914 | -0.08 |
|  | **NO-SOR** | 0.24 (0.13) | 0.26 (0.08) | 39 | 0.668 | -0.15 |

*Note.* Test: 71 of 83 children with a functional scan adequate for analysis had a complete BASC-3 assessment. Following latent profile analysis, 23 children were assigned to the dysregulated cluster and 48 to the resilient cluster. 31 children were SOR and 40 were NO-SOR. Dysregulated: SOR (n = 11, 47.83%), NO-SOR (n = 12, 52.17%); Resilient: SOR (n = 20, 41.67%), NO-SOR (n = 28, 58.33%). Retest: 47 of 71 children possessed both a retest functional scan and BASC-3 evaluation. 15 children were assigned to the dysregulated cluster and 32 to the resilient cluster. 26 children were SOR and 21 were NO-SOR. Dysregulated: SOR (n = 9, 60.00%), NO-SOR (n = 6, 40.00%); Resilient: SOR (n = 17, 53.13%), NO-SOR (n = 15, 46.88%). Summary statistics—including the mean framewise displacement (FD) in mm and standard deviation for each grouping, p-value following Mann-Whitney U tests, and effect size indicated by Cohen’s *d*—are reported. BASC-3, Behavior Assessment System for Children, 3rd edition; SOR, neurodiverse children with sensory over-responsivity; NO-SOR, neurodiverse children without sensory over-responsivity.

Supplementary Table 10.

**2x2x2 ANOVA with factors *SOR status* (SOR, NO-SOR), *Behavior* (DYS, RES), and *Network type* (EXO, ENDO).**

| **FC Metric** | **Set** | ***Effect*** | ***DFn*** | ***DFd*** | ***SSn*** | ***SSd*** | ***F*** | ***p*** | $\boldsymbol{\eta}$***_G_^2^*** |
| --- | --- | --- | --- | --- | --- | --- | --- | --- | --- |
| **Dual Regression** | **Test**  **(n = 71)** | ***(Intercept)*** | 1 | 67 | 0.410 | 43.168 | 0.636 | 0.428 | 0.007 |
|  |  | ***SOR vs. NO-SOR*** | 1 | 67 | 0.705 | 43.168 | 1.094 | 0.299 | 0.013 |
|  |  | ***DYS vs. RES*** | 1 | 67 | 0.778 | 43.168 | 1.207 | 0.276 | 0.014 |
|  |  | ***EXO vs. ENDO*** | 1 | 67 | 0.027 | 11.561 | 0.159 | 0.692 | 0.001 |
|  |  | ***SOR/NO-SOR x DYS/RES*** | 1 | 67 | 0.073 | 43.168 | 0.113 | 0.737 | 0.001 |
|  |  | ***SOR/NO-SOR x EXO/ENDO*** | 1 | 67 | 0.524 | 11.561 | 3.039 | 0.086 | 0.009 |
|  |  | ***DYS/RES x EXO/ENDO*** | 1 | 67 | 0.039 | 11.561 | 0.226 | 0.636 | 0.001 |
|  |  | ***SOR/NO-SOR x DYS/RES x EXO/ENDO*** | 1 | 67 | 0.647 | 11.561 | 3.752 | 0.057 | 0.012 |
|  | **Retest**  **(n = 47)** | ***(Intercept)*** | 1 | 43 | 0.087 | 36.244 | 0.103 | 0.750 | 0.002 |
|  |  | ***SOR vs. NO-SOR*** | 1 | 43 | 0.092 | 36.244 | 0.109 | 0.743 | 0.002 |
|  |  | ***DYS vs. RES*** | 1 | 43 | 0.002 | 36.244 | 0.002 | 0.961 | 0.000 |
|  |  | ***EXO vs. ENDO*** | 1 | 43 | 0.226 | 5.841 | 1.662 | 0.204 | 0.005 |
|  |  | ***SOR/NO-SOR x DYS/RES*** | 1 | 43 | 0.452 | 36.244 | 0.536 | 0.468 | 0.011 |
|  |  | ***SOR/NO-SOR x EXO/ENDO*** | 1 | 43 | 0.629 | 5.841 | 4.633 | **0.037*** | **0.015** |
|  |  | ***DYS/RES x EXO/ENDO*** | 1 | 43 | 0.319 | 5.841 | 2.351 | 0.133 | 0.008 |
|  |  | ***SOR/NO-SOR x DYS/RES x EXO/ENDO*** | 1 | 43 | 1.508 | 5.841 | 11.104 | **0.002**** | **0.035** |
| **fALFF** | **Test**  **(n = 71)** | ***(Intercept)*** | 1 | 67 | 0.045 | 16.494 | 0.182 | 0.671 | 0.001 |
|  |  | ***SOR vs. NO-SOR*** | 1 | 67 | 0.050 | 16.494 | 0.205 | 0.652 | 0.001 |
|  |  | ***DYS vs. RES*** | 1 | 67 | 0.155 | 16.494 | 0.631 | 0.430 | 0.003 |
|  |  | ***EXO vs. ENDO*** | 1 | 67 | 0.013 | 45.025 | 0.019 | 0.891 | 0.000 |
|  |  | ***SOR/NO-SOR x DYS/RES*** | 1 | 67 | 0.145 | 16.494 | 0.589 | 0.445 | 0.002 |
|  |  | ***SOR/NO-SOR x EXO/ENDO*** | 1 | 67 | 0.001 | 45.025 | 0.002 | 0.963 | 0.000 |
|  |  | ***DYS/RES x EXO/ENDO*** | 1 | 67 | 0.065 | 45.025 | 0.097 | 0.756 | 0.001 |
|  |  | ***SOR/NO-SOR x DYS/RES x EXO/ENDO*** | 1 | 67 | 2.323 | 45.025 | 3.457 | 0.067 | 0.036 |
|  | **Retest**  **(n = 47)** | ***(Intercept)*** | 1 | 43 | 0.059 | 13.034 | 0.193 | 0.663 | 0.002 |
|  |  | ***SOR vs. NO-SOR*** | 1 | 43 | 0.122 | 13.034 | 0.401 | 0.530 | 0.003 |
|  |  | ***DYS vs. RES*** | 1 | 43 | 0.125 | 13.034 | 0.413 | 0.524 | 0.003 |
|  |  | ***EXO vs. ENDO*** | 1 | 43 | 0.270 | 24.211 | 0.480 | 0.492 | 0.007 |
|  |  | ***SOR/NO-SOR x DYS/RES*** | 1 | 43 | 0.082 | 13.034 | 0.271 | 0.605 | 0.002 |
|  |  | ***SOR/NO-SOR x EXO/ENDO*** | 1 | 43 | 0.078 | 24.211 | 0.139 | 0.711 | 0.002 |
|  |  | ***DYS/RES x EXO/ENDO*** | 1 | 43 | 0.651 | 24.211 | 1.157 | 0.288 | 0.017 |
|  |  | ***SOR/NO-SOR x DYS/RES x EXO/ENDO*** | 1 | 43 | 2.068 | 24.211 | 3.672 | 0.062 | 0.053 |
| **ReHo** | **Test**  **(n = 71)** | ***(Intercept)*** | 1 | 67 | 0.331 | 18.832 | 1.179 | 0.281 | 0.006 |
|  |  | ***SOR vs. NO-SOR*** | 1 | 67 | 0.044 | 18.832 | 0.156 | 0.694 | 0.001 |
|  |  | ***DYS vs. RES*** | 1 | 67 | 0.399 | 18.832 | 1.421 | 0.237 | 0.007 |
|  |  | ***EXO vs. ENDO*** | 1 | 67 | 0.035 | 35.466 | 0.067 | 0.796 | 0.001 |
|  |  | ***SOR/NO-SOR x DYS/RES*** | 1 | 67 | 0.000 | 18.832 | 0.001 | 0.978 | 0.000 |
|  |  | ***SOR/NO-SOR x EXO/ENDO*** | 1 | 67 | 0.002 | 35.466 | 0.004 | 0.950 | 0.000 |
|  |  | ***DYS/RES x EXO/ENDO*** | 1 | 67 | 0.020 | 35.466 | 0.038 | 0.845 | 0.000 |
|  |  | ***SOR/NO-SOR x DYS/RES x EXO/ENDO*** | 1 | 67 | 1.083 | 35.466 | 2.047 | 0.157 | 0.020 |
|  | **Retest**  **(n = 47)** | ***(Intercept)*** | 1 | 43 | 0.766 | 9.832 | 3.352 | 0.074 | 0.024 |
|  |  | ***SOR vs. NO-SOR*** | 1 | 43 | 0.046 | 9.832 | 0.199 | 0.658 | 0.001 |
|  |  | ***DYS vs. RES*** | 1 | 43 | 0.737 | 9.832 | 3.225 | 0.080 | 0.023 |
|  |  | ***EXO vs. ENDO*** | 1 | 43 | 1.340 | 20.826 | 2.766 | 0.104 | 0.042 |
|  |  | ***SOR/NO-SOR x DYS/RES*** | 1 | 43 | 0.000 | 9.832 | 0.000 | 0.989 | 0.000 |
|  |  | ***SOR/NO-SOR x EXO/ENDO*** | 1 | 43 | 0.256 | 20.826 | 0.528 | 0.471 | 0.008 |
|  |  | ***DYS/RES x EXO/ENDO*** | 1 | 43 | 2.378 | 20.826 | 4.910 | **0.032*** | **0.072** |
|  |  | ***SOR/NO-SOR x DYS/RES x EXO/ENDO*** | 1 | 43 | 2.576 | 20.826 | 5.318 | **0.026*** | **0.077** |

*Note.* Summary of three-way mixed ANOVAs with a 2x2x2 factorial design conducted for each FC metric: dual regression, fALFF, and ReHo within test and retest sets. *SOR status* (SOR, NO-SOR) and *behavior* (dysregulated, resilient) are between-subjects factors, and *network type* (exogenous, endogenous) is a within-subjects factor. We report the effects being examined, degrees of freedom for the numerator (*DFn*) and denominator (*DFd*), sum of squares for the numerator (*SSn*) and denominator (*SSd*), F-value, p-value, and generalized eta squared ($\eta$*_G_^2^*). All values are rounded to three decimal places. FC, functional connectivity; fALFF, fractional amplitude of low-frequency fluctuations; ReHo, regional homogeneity; SOR, neurodiverse children with sensory over-responsivity; NO-SOR, neurodiverse children without sensory over-responsivity; DYS, dysregulated; RES, resilient; EXO, exogenous; ENDO, endogenous.

Supplementary Table 11.

**Comparisons for *Behavior* x *SOR status* x *Network type*.**

| **Set** | **FC Metric** | **Behavior** | **Grouped By** | **Comparison** | ***U*** | **p-value** | **Cohen’s *d*** |
| --- | --- | --- | --- | --- | --- | --- | --- |
| **Test**  **(n = 71)** | **fALFF** | Dysregulated | SOR | Exo vs. Endo | 56 | 0.793 | 0.03 |
|  |  |  | NO-SOR |  | 88 | 0.371 | 0.08 |
|  |  |  | Exogenous | SOR vs. NO-SOR | 63 | 0.878 | -0.15 |
|  |  |  | Endogenous |  | 69 | 0.878 | -0.07 |
|  |  | Resilient | SOR | Exo vs. Endo | 104 | **0.010**** | **-0.95** |
|  |  |  | NO-SOR |  | 531 | **0.023*** | **0.72** |
|  |  |  | Exogenous | SOR vs. NO-SOR | 144 | **0.005**** | **-0.82** |
|  |  |  | Endogenous |  | 403 | **0.010**** | **0.83** |
|  | **ReHo** | Dysregulated | SOR | Exo vs. Endo | 58 | 0.896 | -0.07 |
|  |  |  | NO-SOR |  | 69 | 0.885 | -0.11 |
|  |  |  | Exogenous | SOR vs. NO-SOR | 70 | 0.829 | 0.13 |
|  |  |  | Endogenous |  | 71 | 0.782 | 0.07 |
|  |  | Resilient | SOR | Exo vs. Endo | 120 | **0.032*** | **-0.59** |
|  |  |  | NO-SOR |  | 511 | 0.052 | 0.55 |
|  |  |  | Exogenous | SOR vs. NO-SOR | 191 | 0.064 | -0.51 |
|  |  |  | Endogenous |  | 381 | **0.036*** | **0.62** |
| **Retest**  **(n = 47)** | **fALFF** | Dysregulated | SOR | Exo vs. Endo | 37 | 0.791 | -0.13 |
|  |  |  | NO-SOR |  | 15 | 0.699 | -0.38 |
|  |  |  | Exogenous | SOR vs. NO-SOR | 32 | 0.607 | 0.33 |
|  |  |  | Endogenous |  | 28 | 0.955 | 0.03 |
|  |  | Resilient | SOR | Exo vs. Endo | 95 | 0.091 | -0.53 |
|  |  |  | NO-SOR |  | 191 | **0.001***** | **1.36** |
|  |  |  | Exogenous | SOR vs. NO-SOR | 50 | **0.004**** | **-1.16** |
|  |  |  | Endogenous |  | 179 | 0.054 | 0.72 |
|  | **ReHo** | Dysregulated | SOR | Exo vs. Endo | 33 | 0.536 | -0.35 |
|  |  |  | NO-SOR |  | 11 | 0.310 | -0.88 |
|  |  |  | Exogenous | SOR vs. NO-SOR | 28 | 0.955 | 0.16 |
|  |  |  | Endogenous |  | 26 | 0.955 | -0.38 |
|  |  | Resilient | SOR | Exo vs. Endo | 98 | 0.113 | -0.48 |
|  |  |  | NO-SOR |  | 193 | **0.001***** | **1.51** |
|  |  |  | Exogenous | SOR vs. NO-SOR | 44 | **0.002**** | **-1.33** |
|  |  |  | Endogenous |  | 183 | **0.038*** | **0.73** |

*Note.* The groupings and comparisons involve *behavior* (dysregulated, resilient), *SOR status* (SOR, NO-SOR), and *network type* (exogenous, endogenous). The p-value following post hoc Mann-Whitney U tests and effect size indicated by Cohen’s *d* are reported. Statistical significance is indicated by asterisks: * *p* ≤ 0.05, ** *p* ≤ 0.01, and *** *p* ≤ 0.001. FC, functional connectivity; fALFF, fractional amplitude of low-frequency fluctuations; ReHo, regional homogeneity; SOR, neurodiverse children with sensory over-responsivity; NO-SOR, neurodiverse children without sensory over-responsivity; EXO, exogenous; ENDO, endogenous.

Supplementary Table 12.

**Comparisons for *SOR status* x *Behavior* x *Network type*.**

| **Set** | **FC Metric** | **SOR status** | **Grouped By** | **Comparison** | ***U*** | **p-value** | **Cohen’s *d*** |
| --- | --- | --- | --- | --- | --- | --- | --- |
| **Test**  **(n = 71)** | **fALFF** | SOR | Dysregulated | Exo vs. Endo | 56 | 0.793 | 0.03 |
|  |  |  | Resilient |  | 104 | **0.010**** | **-0.95** |
|  |  |  | Exogenous | Dys vs. Res | 146 | 0.143 | 0.57 |
|  |  |  | Endogenous |  | 92 | 0.470 | -0.39 |
|  |  | NO-SOR | Dysregulated | Exo vs. Endo | 88 | 0.371 | 0.08 |
|  |  |  | Resilient |  | 531 | **0.023*** | **0.72** |
|  |  |  | Exogenous | Dys vs. Res | 154 | 0.690 | -0.16 |
|  |  |  | Endogenous |  | 208 | 0.244 | 0.50 |
|  | **ReHo** | SOR | Dysregulated | Exo vs. Endo | 58 | 0.896 | -0.07 |
|  |  |  | Resilient |  | 120 | **0.032*** | **-0.59** |
|  |  |  | Exogenous | Dys vs. Res | 136 | 0.292 | 0.49 |
|  |  |  | Endogenous |  | 97 | 0.606 | -0.07 |
|  |  | NO-SOR | Dysregulated | Exo vs. Endo | 69 | 0.885 | -0.11 |
|  |  |  | Resilient |  | 511 | 0.052 | 0.55 |
|  |  |  | Exogenous | Dys vs. Res | 147 | 0.545 | -0.16 |
|  |  |  | Endogenous |  | 209 | 0.232 | 0.48 |
| **Retest**  **(n = 47)** | **fALFF** | SOR | Dysregulated | Exo vs. Endo | 37 | 0.791 | -0.13 |
|  |  |  | Resilient |  | 95 | 0.091 | -0.53 |
|  |  |  | Exogenous | Dys vs. Res | 94 | 0.360 | 0.45 |
|  |  |  | Endogenous |  | 71 | 0.788 | 0.00 |
|  |  | NO-SOR | Dysregulated | Exo vs. Endo | 15 | 0.699 | -0.38 |
|  |  |  | Resilient |  | 191 | **0.001***** | **1.36** |
|  |  |  | Exogenous | Dys vs. Res | 23 | 0.095 | -1.12 |
|  |  |  | Endogenous |  | 63 | 0.178 | 0.71 |
|  | **ReHo** | SOR | Dysregulated | Exo vs. Endo | 33 | 0.536 | -0.35 |
|  |  |  | Resilient |  | 98 | 0.113 | -0.48 |
|  |  |  | Exogenous | Dys vs. Res | 83 | 0.746 | 0.36 |
|  |  |  | Endogenous |  | 92 | 0.419 | 0.27 |
|  |  | NO-SOR | Dysregulated | Exo vs. Endo | 11 | 0.310 | -0.88 |
|  |  |  | Resilient |  | 193 | **0.001***** | **1.51** |
|  |  |  | Exogenous | Dys vs. Res | 21 | 0.066 | -1.02 |
|  |  |  | Endogenous |  | 76 | **0.014*** | **1.40** |

*Note.* The groupings and comparisons involve *SOR status* (SOR, NO-SOR), *behavior* (dysregulated, resilient), and *network type* (exogenous, endogenous). The p-value following post hoc Mann-Whitney U tests and effect size indicated by Cohen’s *d* are reported. Statistical significance is indicated by asterisks: * *p* ≤ 0.05, ** *p* ≤ 0.01, and *** *p* ≤ 0.001. FC, functional connectivity; fALFF, fractional amplitude of low-frequency fluctuations; ReHo, regional homogeneity; SOR, neurodiverse children with sensory over-responsivity; NO-SOR, neurodiverse children without sensory over-responsivity; DYS, dysregulated; RES, resilient; EXO, exogenous; ENDO, endogenous.

**References**

112. Tustison NJ, Avants BB, Cook PA, Zheng Y, Egan A, Yushkevich PA, et al. N4ITK: improved N3 bias correction. IEEE Trans Med Imaging. 2010;29(6):1310–20.

113. Avants BB, Epstein CL, Grossman M, Gee JC. Symmetric diffeomorphic image registration with cross-correlation: evaluating automated labeling of elderly and neurodegenerative brain. Med Image Anal. 2008;12(1):26–41.

114. Klein A, Ghosh SS, Bao FS, Giard J, Häme Y, Stavsky E, et al. Mindboggling morphometry of human brains. PLoS Comput Biol. 2017;13(2):e1005350.

115. Andersson JLR, Skare S, Ashburner J. How to correct susceptibility distortions in spin-echo echo-planar images: application to diffusion tensor imaging. Neuroimage. 2003;20(2):870–88.

116. Jenkinson M, Bannister P, Brady M, Smith S. Improved optimization for the robust and accurate linear registration and motion correction of brain images. NeuroImage. 2002;17(2):825–41.

117. Greve DN, Fischl B. Accurate and robust brain image alignment using boundary-based registration. Neuroimage. 2009;48(1):63–72.

118. Power JD, Mitra A, Laumann TO, Snyder AZ, Schlaggar BL, Petersen SE. Methods to detect, characterize, and remove motion artifact in resting state fMRI. Neuroimage. 2014;84:320–41.

119. Behzadi Y, Restom K, Liau J, Liu TT. A component based noise correction method (CompCor) for BOLD and perfusion based fMRI. Neuroimage. 2007;37(1):90–101.

120. Satterthwaite TD, Elliott MA, Gerraty RT, Ruparel K, Loughead J, Calkins ME, et al. An improved framework for confound regression and filtering for control of motion artifact in the preprocessing of resting-state functional connectivity data. NeuroImage. 2013;64:240–56.

121. Patriat R, Reynolds RC, Birn RM. An improved model of motion-related signal changes in fMRI. Neuroimage. 2017;144(Pt A):74–82.

122. Lanczos C. Evaluation of noisy data. J Soc Industrial Appl Math Ser B Numer Anal. 1964;1(1):76–85.
